# Dual CAR-NK cells targeting PD-L1 and ErbB2 (HER2) exhibit cooperative CAR signaling and counteract solid tumor heterogeneity

**DOI:** 10.1186/s13046-026-03722-6

**Published:** 2026-05-19

**Authors:** Kristin Freudenberg-Jahn, Idan Ben-Horin, Nivedha Murali Shankar, Maria Schuldt, Paola Ortiz-Montero, Liliana R. Loureiro, Wiebke Rackwitz, Corinna Opitz, Lydia Krutz, Thomas Breither, Pranav Oberoi, Aline Häcker, Dirk Jäger, Jan Dominik Kuhlmann, Pauline Wimberger, Stephan R. Künzel, Kristina Hölig, Anja Feldmann, Michael Bachmann, Winfried S. Wels, Torsten Tonn, Jiri Eitler

**Affiliations:** 1 Experimental Transfusion Medicine, Faculty of Medicine Carl Gustav Carus, Dresden University of Technology, Dresden, Germany; 2 Institute for Transfusion Medicine Dresden, German Red Cross Blood Donation Service North-East, Dresden, Germany; 3 German Cancer Consortium (DKTK), Partner Site Dresden, Dresden, Germany; 4 Georg-Speyer-Haus, Institute for Tumor Biology and Experimental Therapy, Frankfurt am Main, Germany; 5 Department of Radioimmunology, Institute of Radiopharmaceutical Cancer Research, Helmholtz-Zentrum Dresden-Rossendorf, Dresden, Germany; 6 Department of Medical Oncology, National Center for Tumor Diseases Heidelberg, University Hospital Heidelberg, Heidelberg, Germany; 7 National Center for Tumor Diseases (NCT), University Hospital Carl Gustav Carus, TU Dresden, Dresden, Germany; 8 Tumor Immunology, University Cancer Center (UCC), University Hospital Carl Gustav Carus, TU Dresden, Dresden, Germany; 9 Frankfurt Cancer Institute, Goethe University, Frankfurt am Main, Germany; 10 Department of Gynecology and Obstetrics, Medical Faculty and University Hospital Carl Gustav Carus, Technische Universität Dresden, Dresden, Germany; 11 National Center for Tumor Diseases (NCT), NCT/UCC Dresden, a Partnership Between DKFZ, Faculty of Medicine and University Hospital Carl Gustav Carus, TUD Dresden University of Technology, and Helmholtz-Zentrum Dresden-Rossendorf (HZDR), Dresden, Germany; 12 Dresden and German Cancer Research Center (DKFZ), German Cancer Consortium (DKTK), Heidelberg, Germany; 13 German Red Cross Blood Donation Service Baden-Württemberg-Hessen gGmbH, Mannheim, Germany; 14 Institute for Transfusion Medicine and Immunohematology, Clinics of the Goethe University, Frankfurt am Main, Germany

**Keywords:** NK cells, NK-92, Chimeric antigen receptor (CAR), Dual CAR, PD-L1, ErbB2 (HER2), Cancer immunotherapy, Therapy resistance, Solid tumors, Primary human tumors

## Abstract

**Background:**

Chimeric antigen receptor (CAR)-engineered natural killer (NK) cells have shown potent efficacy against hematologic malignancies. In contrast, solid tumors remain difficult to treat due to heterogeneous antigen expression and an immunosuppressive tumor microenvironment that fosters immune evasion and is driven to a large part by checkpoint-mediated inhibition.

**Methods:**

To overcome these barriers, we engineered novel dual CAR-NK cells targeting the immune checkpoint PD-L1 and the tumor-associated antigen ErbB2 (HER2). The clinically applicable NK-92 cell line was lentivirally transduced with PD-L1.CAR and ErbB2.CAR constructs. Antitumor activity was assessed across diverse solid tumor models using a comprehensive panel of in vitro and in vivo assays.

**Results:**

Dual CAR-NK cells exhibited potent and selective cytotoxicity against breast, pancreatic, gastric, and lung cancer cell lines expressing either one or both targets. Comparable efficacy was observed in 3D spheroid cultures and against patient-derived primary ovarian cancer cells. Notably, dual CAR-NK cells retained strong cytotoxicity when one antigen was absent or blocked, modeling immune escape through antigen loss - a feature absent in single CAR-NK cells. Mechanistically, each CAR independently activated the MEK/ERK pathway in an antigen-dependent manner, while dual CAR stimulation potentiated the signaling response in a cooperative manner. Furthermore, IFN-γ produced by NK cells upon encounter of resistant cancer cells was sufficient to induce PD-L1 upregulation, thereby enhancing the potency of the dual CAR system. In a PD-L1/ErbB2 double-positive breast cancer xenograft model, dual CAR-NK cells consistently outperformed single-target controls.

**Conclusions:**

Dual targeting of PD-L1 and ErbB2 enhances CAR-NK cell efficacy against refractory solid tumors by providing resilience to antigen heterogeneity and amplifying antitumor signaling through cooperative activation. This approach represents a promising and adaptable platform for clinical translation in solid tumor immunotherapy.

**Graphical Abstract:**

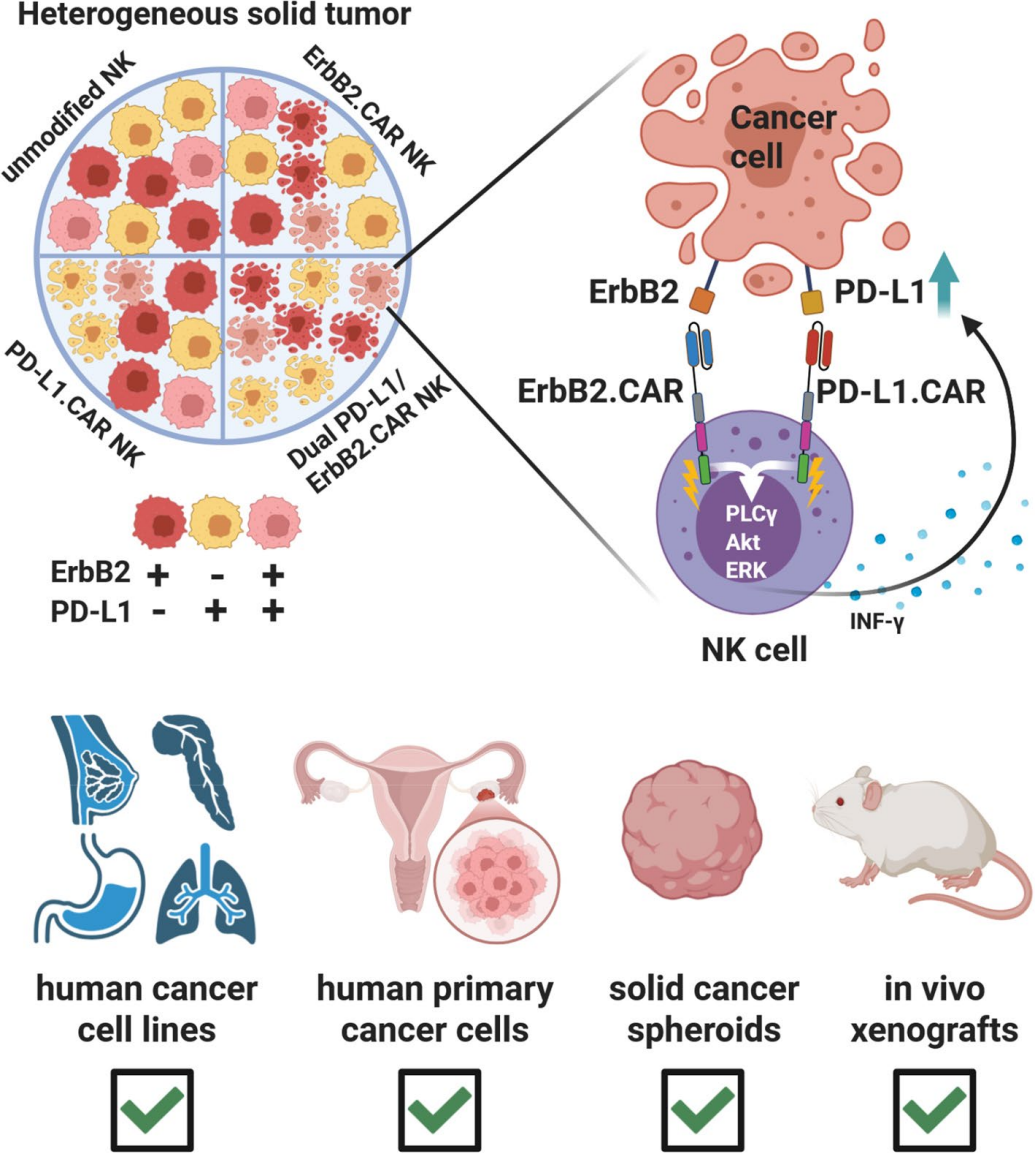

**Supplementary Information:**

The online version contains supplementary material available at 10.1186/s13046-026-03722-6.

## Highlights

Dual targeting of PD-L1 and ErbB2 enhances CAR-NK cell efficacy against different heterogeneous solid tumor models, including primary ovarian cancer and in vivo xenografts.

Dual PD-L1/ErbB2 CAR NK cells counteract immune escape due to antigen loss.

Dual stimulation by PD-L1 and ErbB2 leads to enhanced signaling through the ERK1/2 pathway.

Activated NK cells stimulate PD-L1 upregulation on cancer cells through soluble factors.

**Supplementary Information:**

The online version contains supplementary material available at 10.1186/s13046-026-03722-6.

## Introduction

Chimeric antigen receptor (CAR)-engineered T cells and antibodies which block important immune checkpoints have revolutionized cancer therapy [1, 2], and combining these two strategies may further improve treatment success [3, 4]. Nevertheless, limitations of current CAR-T cell approaches still pose a challenge to the field. While CAR-T cells are an effective therapy for hematological malignancies of the B-cell lineage, serious side effects such as cytokine release syndrome (CRS) and immune effector cell-associated neurotoxicity syndrome (ICANS) complicate treatment [5]. Furthermore, the presence of a T-cell receptor (TCR) of unknown specificity poses the risk of severe graft-versus-host disease (GvHD) when using unaltered allogeneic T cells for adoptive transfer, which complicates the development of readily available off-the-shelf products. In contrast to T lymphocytes, natural killer (NK) cells can be safely applied in an allogeneic setting without a relevant risk of inducing GvHD [6]. As part of the innate immune system, NK cells are cytotoxic lymphocytes that do not express a TCR and are not MHC-restricted, but recognize cancer cells through a variety of germline-encoded activating receptors [7]. Hence, they can respond rapidly to specific signals without prior sensitization or clonal selection. This makes them attractive effector cells for the development of cellular immunotherapies that can readily be made available to patients in a cost-effective manner and independent from the patients’ own immune competence [8].

NK cell products and CAR-engineered variants derived from sources such as peripheral blood of healthy donors, cord blood, or differentiated from hematopoietic or induced pluripotent stem (iPS) cells are under active development [9–11]. Early clinical data have confirmed safety and efficacy of adoptively transferred cord blood-derived CAR-NK cells in patients with CD19-positive B-cell leukemia or lymphoma, without causing side effects like CRS or neurotoxicity [12, 13]. Alternatively, stable NK cell lines are considered for clinical use, with safety and clinical activity of continuously expanding NK-92 cells shown in early phase clinical trials [14–18]. NK-92 cells have also been instrumental to transfer the concept of CAR-engineering from T cells to NK cells [19]. Numerous studies meanwhile demonstrated high efficacy of CAR NK-92 cells in preclinical models of hematological malignancies and solid tumors [20–23], and initial phase I clinical trials established safety and feasibility of such approaches for the treatment of cancer patients [24, 25]. We previously generated a clinical-grade CAR NK-92 cell line (NK-92/5.28.z; hereafter termed ErbB2.CAR NK-92) targeting the tumor-associated antigen ErbB2 (HER2) [26], a receptor tyrosine kinase expressed at enhanced levels by many cancers of solid tumor origin [27]. The retargeted ErbB2.CAR NK-92 cells demonstrated high selectivity and efficacy in preclinical models of different cancer types [28, 29], thereby overcoming NK-cell resistance of tumor cells by restoring signaling pathways crucial for lytic granule polarization and degranulation [30, 31]. At present, the ErbB2.CAR NK-92 cells are evaluated in an ongoing phase I clinical trial in patients with recurrent ErbB2-positive glioblastoma (CAR2BRAIN; NCT03383978) [24].

Nevertheless, targeting solid tumors with CAR-engineered effector cells remains challenging. Thereby, the often observed heterogeneity of target antigen expression within the tumor represents an important problem, since therapy-induced selection of antigen-loss variants can lead to treatment resistance and tumor immune escape [32, 33]. One approach to overcome this limitation is to increase the therapeutic bandwidth by targeting multiple tumor-associated antigens in parallel, using either pooled CAR effector populations or dual/multispecific CAR constructs within single cells [34, 35]. Another challenge is the immunosuppressive microenvironment of many solid tumors, which can lead to exhaustion of effector cells or even prevent induction of productive anti-tumor immune responses. Immune checkpoint inhibitors, in particular those targeting PD-1 on T cells or its ligand PD-L1 on cancer cells can counteract inhibitory signals and reverse T-cell exhaustion, resulting in improved anti-tumor responses [2]. PD-L1 has also been suggested as a target for CAR-engineered T and NK cells [21, 34, 36]. However, only a fraction of cancer cells within the tumor may express PD-L1, or PD-L1 levels on the cell surface may be very low, limiting the potential benefit of CAR effector cells solely directed to PD-L1 [37].

Based on clinical-grade ErbB2.CAR NK-92 cells, here we generated by additional genetic modification novel CAR-engineered NK cell variants targeting both, ErbB2 and PD-L1. Antitumoral activity of the resulting dual CAR-NK cells in comparison to respective monospecific NK-92 cells was analyzed in in vitro assays with ErbB2- and/or PD-L1-positive cancer cell lines of various solid tumor origins growing in 2D culture or as 3D spheroids, as well as with patient-derived primary ovarian carcinoma cells. Efficacy of the dual CAR-NK cells in vivo was investigated in a breast carcinoma xenograft model.

## Materials and methods

### Cells and cell culture

The established human NK cell line NK-92 was kindly provided by H.G. Klingemann (Vancouver, Canada) [38]. NK-92/5.28.z cells engineered with an ErbB2-specific CAR were previously generated as described [28]. The NK cell lines were cultured in X-VIVO 10 medium (Lonza) containing 5% heat-inactivated human AB plasma (German Red Cross Blood Donation Service North-East, Dresden, Germany), 500 IU/mL IL-2 (Proleukin; Novartis Pharma). Human MDA-MB-231, MDA-MB-453, MCF-7, PANC-1, BxPC-3, MIA PaCa-2, KATO III, NCI-N87, A549, Calu-3 and murine B16F10 cells were purchased from the American Type Culture Collection (ATCC; Manassas, VA, USA). MDA-MB-453/PD-L1 and MDA-MB-468/PD-L1 cells were kindly provided by I. Kühnel and J. Pfeifer Serrahima (Frankfurt am Main, Germany), B16F10/ErbB2 cells [39] were kindly provided by M. Kershaw (Melbourne, Australia). BxPC-3, KATO III and NCI-N87 cells were cultured in RPMI 1640 medium (Merck/Biochrom) supplemented with 10% heat-inactivated fetal bovine serum (HI-FBS; Merck/Biochrom), 2 mM L-glutamine (Merck/Biochrom), 100 IU/mL penicillin, and 100 μg/mL streptomycin. MDA-MB-231, MDA-MB-453, MCF-7, PANC-1, MIA PaCa-2, A549, Calu-3, B16F10 and B16F10/ErbB2 cells were cultured in DMEM medium (Merck/Biochrom) supplemented with 10% HI-FBS, 2 mM L-glutamine, 100 IU/mL penicillin, and 100 μg/mL streptomycin. Medium for MCF-7 cells was additionally supplemented with 10 µg/mL insulin (Sigma-Aldrich). All cells were cultured at 37 °C in a humidified atmosphere with 5% CO_2_ and routinely checked for Mycoplasma contamination.

Human primary NK cells were isolated from healthy donors in accordance with protocols approved by the local ethics committee. Peripheral blood mononuclear cells (PBMCs) were obtained from buffy coats by Biocoll density gradient centrifugation (Merck/Biochrom), and NK cells were isolated by negative selection using the NK Cell Isolation Kit and autoMACS system (Miltenyi Biotec) according to the manufacturer’s instructions. Isolated NK cells were cultured in NK MACS medium (Miltenyi Biotec) supplemented with 5% human AB serum (German Red Cross Blood Donation Service North-East), 1000 IU/mL IL-2, and 20 ng/mL IL-21 (Miltenyi Biotec). PBMCs and mesenchymal stem cells (MSCs) used for off-tumor cytotoxicity analysis were obtained from healthy donors in accordance with protocols approved by the local ethics committee (German Red Cross Blood Donation Service North-East, Dresden, Germany). PBMCs were isolated from buffy coats as described above and cryopreserved. MSCs were isolated from human bone marrow by Ficoll density gradient centrifugation to obtain mononuclear cells, followed by plastic adherence. Cells were cultured in DMEM supplemented with 5% fetal bovine serum, and non-adherent cells were removed after 24 h by washing. Adherent MSCs were expanded with medium changes twice weekly and cryopreserved until use. Human primary ovarian cancer ascites samples were obtained from female patients with ovarian cancer and subsequently cryopreserved. After thawing, the cells were washed and used directly in cytotoxicity co-culture assays with CAR-NK cells. For the Europium-based cytotoxicity assay, primary ovarian cancer cells were isolated from malignant ascites of a female patient with HER2-positive ovarian cancer. Tumor cells were enriched from the ascites by immunomagnetic separation using the Tumor Cell Isolation Kit according to the manufacturer’s instructions (Miltenyi Biotec).

### Flow cytometry

Cells were stained for 30 min on ice with antibodies specific for ErbB2 (191,924; R&D Systems) and PD-L1 (MIH1, BD Biosciences). ErbB2-CAR detection was performed using an ErbB2-Fc fusion protein (R&D Systems), followed by staining with anti-human Fc secondary antibody (Jackson Immunoresearch) as previously described [26]. Likewise, PD-L1-CAR expression was detected using PD-L1-Fc (BioLegend). Live cells were discriminated using 7-AAD (BD Biosciences). For phosphorylation experiments, cells were fixed with BD Cytofix, permeabilized with BD Perm Buffer III (BD Biosciences), and stained with rabbit anti-pAkt, anti-pPLCγ1 and anti-pERK1/2 antibodies (Cell Signaling), followed by goat anti-rabbit secondary antibody (Thermo Fisher Scientific). Samples were acquired using a BD FACSCanto II flow cytometer and data were analyzed using FlowJo software version 9 (BD Biosciences).

### Generation of transgenic cells

PD-L1/ErbB2.CAR and PD-L1.CAR NK-92 cells were generated by lentiviral transduction of ErbB2.CAR NK-92 (NK-92/5.28.z) or parental NK-92 cells, respectively, with a second-generation CAR consisting of a PD-L1-specific scFv fragment, a CD8α hinge region, CD28 transmembrane/costimulatory, and CD3ζ intracellular signaling domains, cloned into pSIEW (PD-L1.CAR) or pSIRW (PD-L1.CAR2) vector backbones as described previously [28, 40, 41]. ScFv antibody sequences for PD-L1.CAR and atezolizumab-based PD-L1.CAR2 were derived from [21] and [42]. PD-L1.CAR-positive cells were immunomagnetically enriched according to the manufacturer’s protocol (Miltenyi Biotec) using human recombinant PD-L1-Fc combined with biotinylated anti-human Fc. PD-L1.CAR2-positive cells were selected by flow cytometric cell sorting according to their expression of the vector-encoded iRFP marker protein. Enriched cells yielded > 95% purity of PD-L1.CAR and PD-L1.CAR2 expressing NK-92 cells. Primary NK cells were transduced 4 days post-isolation, and CAR expression was assessed 72 h post-transduction by flow cytometry. ErbB2.CAR expression was detected using an anti-mouse Fab antibody (Jackson ImmunoResearch). PD-L1.CAR expression was analyzed using recombinant human PD-L1-Fc (BioLegend), followed by staining with an anti-human IgG antibody (Jackson ImmunoResearch) after Fc receptor blocking (BD Biosciences). Subsequently, cells were co-stained with anti-CD3 and anti-CD56 antibodies (BD Biosciences). To generate MDA-MB-231 NR, MDA-MB-453 NR, BxPC-3 NR and PANC-1 NR cells, the respective parental cell lines were transduced with Nuclight Red lentiviral particles (Sartorius) at an MOI < 1 by 30 min spinoculation at 1000 × g in the presence of 8 µg/ml Polybrene (Sigma-Aldrich), followed by puromycin selection (Sigma-Aldrich). For in vivo xenograft experiments, MDA-MB-231 cells were transduced with a firefly luciferase (Luc) construct according to the previously described protocol [43]. The resulting MDA-MB-231/Luc cells were further transduced with lentiviral pHAGE-ERBB2 vector to generate MDA-MB-231/Luc/ErbB2 cells. The pHAGE-ERBB2 transfer plasmid was a gift from Gordon Mills and Kenneth Scott (Addgene plasmid #116,734) [44].

### Lentivirus production

Lentiviral particles were produced in HEK293T cells using the psPAX2 and pMD2.G (both from Addgene) or pCMVΔR8.91 and pMD2.G packaging vectors [45]. Plasmids were transfected with polyethyleneimine (PEI) and supernatants were harvested after 48 h. Viral vectors were concentrated with PEG-it solution (System Biosciences) according to the manufacturer's instructions.

### Europium-TDA (EuTDA) cytotoxicity assay

Specific cytotoxicity of NK cells against target cells was determined using an Europium (EuTDA) cytotoxicity assay (DELFIA; PerkinElmer) according to the manufacturer’s protocol. Briefly, target cells were loaded with an acetoxymethyl ester of the fluorescence-enhancing ligand (BATDA; Perkin Elmer) and then co-incubated in triplicates at 10,000 cells/well with effector cells at the indicated E:T ratios. For blocking studies, NK cells were incubated with blocking antibodies prior to mixing with cancer cells as described below. After 2 h of co-culture, supernatants were collected for measurement of the fluorescent signal reflecting target cell lysis using a VICTOR X4 fluorometer (PerkinElmer). Specific lysis was calculated according to the standard formula:

$$\% \text{Specific release}=\frac{\text{Experimental release} \left(\mathrm{counts}\right)-\text{Spontaneous release} \left(\mathrm{counts}\right)}{\text{Maximum release} \left(\mathrm{counts}\right)-\text{Spontaneous release} \left(\mathrm{counts}\right)}\times 100$$

### Flow cytometry based cytotoxicity assay

Alternatively, specific cytotoxicity of NK cells was determined using a FACS-based assay as described previously (*28*). Briefly, tumor cells were labeled with calcein violet AM (CV) (Invitrogen, Thermo Fisher Scientific) and incubated in triplicates with effector cells at the indicated E:T ratios for 2 h at 37 °C. Then 150 µL of a 1 µg/mL propidium iodide (PI) solution were added to each sample before flow cytometric analysis with a FACSCanto II device (BD Biosciences). Dead target cells were identified as CV and PI double positive. Spontaneous target cell lysis was subtracted to calculate specific cytotoxicity.

For primary ovarian cancer ascites, tumor cells were co-cultured with NK cells for 6 h at 37 °C, followed by staining of the mixed cells for ErbB2, PD-L1, and the NK cell marker CD56. Data were gated on DAPI-negative and CD56-negative populations to exclude dead cells and NK cells. Flow cytometric analysis was performed using a BD FACSLyric device (BD Biosciences). Data were analyzed using FlowJo software version 10.6.0 (BD Biosciences).

### Live cell imaging cytotoxicity assays

Live cell imaging cytotoxicity assays were performed using the IncuCyte S3 instrument (Sartorius) according to the manufacturer's protocol. Briefly, 1 × 10^4^ target cells transduced with Nuclight Red fluorescent protein (Sartorius) were seeded in triplicates in a poly-L-ornithine (Sigma-Aldrich)-coated 96-well plate and incubated for 90 min at 37 °C. Next, NK cells were added at a 1:1 E:T ratio and cells were co-cultured in complete X-VIVO 10 medium for 72 h. Images were acquired every 12 h by IncuCyte software. Cytotoxicity was calculated as the confluence of Nuclight Red labeled cancer cells. Data were analyzed using IncuCyte software and normalized to baseline.

### Spheroid cytotoxicity assays

Spheroid cytotoxicity assays were performed using the IncuCyte S3 instrument as described above, with the following modification. Spheroids were generated from 1 × 10^3^ cancer cells in low-adhesion 96-well round bottom plates (Corning) in the presence of 2.5% Matrigel (Corning). Spheroids were grown for 48 h, followed by addition of 1 × 10^3^ NK cells and continued culture for another 120 h at 37°C. Images were acquired every 12 h by IncuCyte software. Cytotoxicity was calculated based on Nuclight Red total integrated intensity using IncuCyte software and normalized to baseline.

### Cytokine measurements

NK and cancer cells were co-cultured in duplicates at an E:T ratio of 1:1 at a density of 5 × 10^5^ cells/mL for each cell type. After 6 h, cells were spun down and supernatants were used for cytokine measurements using the Luminex assay (Procartaplex, Thermofisher) according to the manufacturer's protocols. Measurements were performed on the Luminex Flexmap 3D system.

### IFN-γ stimulation

Cancer cells were incubated with 20 ng/mL human recombinant IFN-γ (BioLegend) for 48 h. Cells were washed, phenotyped by flow cytometry and used for cytotoxicity assays.

### Cytokine neutralization assays

Nuclight Red expressing MDA-MB-453 cells were co-cultured with ErbB2.CAR NK-92 effector cells at an E:T ratio of 1:10. ErbB2.CAR NK-92 cells were pre-incubated for 30 min with cytokine-neutralizing antibodies against TNF-α (Ultra-LEAF™, #648,827, BioLegend) and IFN-γ (Ultra-LEAF™, #506,532, BioLegend) at a concentration of 20 µg/mL, or with corresponding isotype controls (Ultra-LEAF™ purified human IgG1, #506,532, and mouse IgG1, #400,166; BioLegend). Target cells were subsequently added, resulting in a final antibody concentration of 10 µg/mL. After 16 h of co-culture, cells were stained for PD-L1 and CD56 (BD Biosciences) and analyzed by flow cytometry.

### Degranulation and signaling inhibition assays

Effector cells were pretreated for 60 min with pharmacological inhibitors at 2 × the indicated final concentrations (PD0325901 and trametinib, 50 µM; U73122, 5 µM; wortmannin, 20 µM; all from Selleckchem) or corresponding vehicle controls (DMSO or DMF; Sigma-Aldrich). Target cells and anti-CD107a antibody (eBioH4A3, Invitrogen) were then added. After 90 min of incubation, cells were placed on ice, followed by staining with anti-CD56 antibody (BD Biosciences) and analysis by flow cytometry.

### Transwell experiments

The paracrine effect of CAR-NK cells on the expression of PD-L1 in A549 lung cancer cells was studied using transwell inserts (Greiner Bio-One) that allow diffusion of soluble factors. Briefly, 5 × 10^4^ A549 cells were seeded in 600 µL of DMEM in the lower chambers of a 24-well transwell plate. Subsequently, 1.5 × 10^5^ NK cells were transferred to the upper chambers of the transwell plates alone or with A549 tumor cells at an E:T ratio of 1:1. Where indicated, A549 cells were treated with human IFN-γ (50 ng/mL, PeproTech). After 48 h of incubation at 37 °C, the transwell upper chambers were removed, and the A549 tumor cells in the lower chambers were analyzed for PD-L1 expression by flow cytometry with fluorochrome-conjugated antibody (MIH2, BioLegend).

### In vivo xenograft mouse experiments

In vivo experiments involving optical imaging of experimental mice were performed in accordance with the guidelines of the German Regulations for Animal Welfare. The health status of the mice was monitored daily by husbandry staff. After completing all experiments, the animals were euthanized using carbon dioxide inhalation and cervical dislocation. The protocol was approved by the local Ethics Committee for Animal Experiments (AZ DD24.1–5131/449/67). A detailed description of experimental procedures was reported previously [46]. Briefly, 8-weeks-old female NXG mice (NOD-Prkdcscid-IL2rgTm1/Rj; Janvier Labs) were divided into groups of 5 mice. Each group was injected with 1 × 10^6^ MDA-MB-231/Luc/ErbB2 cells in combination with 0.5 × 10^6^ of either NK-92 cells, PD-L1.CAR NK-92 cells, ErbB2.CAR NK-92 cells, or PD-L1/ErbB2.CAR NK-92 cells. Cell suspensions were premixed with Matrigel (1:1; Corning Life Sciences) and injected subcutaneously into the right flank of the mice. For optical imaging, mice were anesthetized as previously described [47], and bioluminescence signals were measured with an IVIS Spectrum In Vivo Imaging System (PerkinElmer). Data were analyzed using Living Image software (PerkinElmer).

### Statistical analysis

Statistical analyses were performed with GraphPad Prism 9 (Graphpad Software). Unpaired two-tailed Student’s t-test was used for statistical calculations unless otherwise stated in the figure legend. A p-value < 0.05 was considered statistically significant. ****, *p* < 0.0001; ***, *p* < 0.001; **, *p* < 0.01; *, *p* < 0.05; ns (not significant) *p* ≥ 0.05.

## Results

### Generation of dual PD-L1/ErbB2-targeted CAR NK-92 cells

**Fig. 1:**
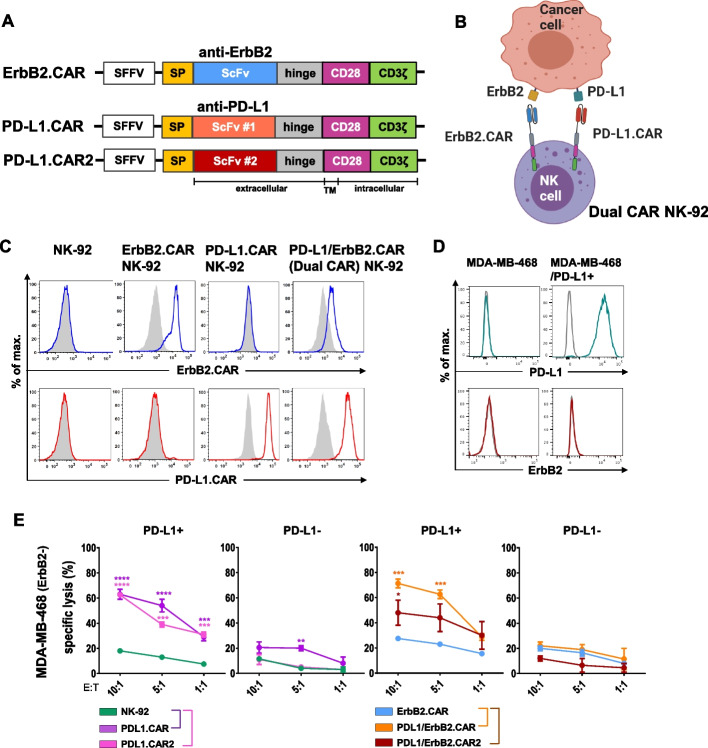
Generation of dual-specific PD-L1/ErbB2.CAR NK-92 cells. **A** Schematic representation of lentiviral constructs encoding ErbB2- and PD-L1-specific CARs under control of the spleen focus-forming virus (SFFV) promoter. Each CAR consists of an immunoglobulin heavy chain signal peptide (SP), an ErbB2- or PD-L1-specific scFv antibody fragment, a CD8α hinge region, the transmembrane and intracellular domains of CD28, and the intracellular domain of CD3ζ. **B** Schematic representation of the dual PD-L1/ErbB2.CAR NK-92 cells generated by lentiviral transduction of ErbB2.CAR NK-92 cells with a PD-L1.CAR vector. Control PD-L1.CAR NK-92 cells were generated by transduction of parental NK-92 with a PD-L1.CAR (not shown). **C** CAR expression on PD-L1/ErbB2.CAR, PD-L1.CAR and ErbB2.CAR NK-92 cells was determined by flow cytometry using recombinant ErbB2-Fc (upper panels) or PD-L1-Fc protein (lower panels), followed by staining with an anti-Fc secondary antibody. Parental NK-92 cells were included for comparison. Filled gray areas indicate negative controls only stained with secondary antibody. Representative data from at least 3 independent experiments are shown. **D** Surface expression of PD-L1 (upper panels) and absence of ErbB2 (lower panels) on MDA-MB-468/PD-L1 breast carcinoma cells modified to ectopically overexpress PD-L1 was confirmed by flow cytometry with fluorochrome-labeled PD-L1- and ErbB2-specific antibodies. PD-L1-negative parental cells were included for comparison. Unstained cells served as controls (gray lines). **E** Cell killing activity of PD-L1.CAR, PD-L1.CAR2, ErbB2.CAR, PD-L1/ErbB2.CAR and PD-L1/ErbB2.CAR2 NK-92 cells against ErbB2- and PD-L1-negative MDA-MB 468 (PD-L1-) and ErbB2-negative but PD-L1-positive MDA-MB-468/PD-L1 cells (PD-L1 +) was investigated in flow cytometry-based cytotoxicity assays at the indicated effector to target (**E**:T) ratios after 2 h of co-culture. Parental NK-92 cells were included as control. Mean values ± SEM are shown; n = 3 independent experiments. ****, *p* < 0.0001; ***, *p* < 0.001; **, *p* < 0.01

We previously generated CAR NK-92 cells specific for ErbB2 (NK-92/5.28.z; here referred to as ErbB2.CAR NK-92), and demonstrated their in vitro and in vivo efficacy against various solid tumor entities in preclinical models [28, 29]. A master cell bank of the ErbB2.CAR NK-92 cells was derived under GMP conditions [26], and the cells are currently evaluated in a phase I clinical trial in patients with recurrent ErbB2-positive glioblastoma [24]. To overcome therapy failures caused by antigen loss and eliminate immunosuppressive tumor cells, here we aimed to further improve the ErbB2.CAR NK-92 cells by a second step of genetic engineering with a CAR targeting the PD-L1 checkpoint molecule (PD-L1.CAR). PD-L1.CAR constructs consisting of single chain fragment variable (scFv) domains derived from one of two different anti-PD-L1 antibodies [21, 42], a CD8α hinge region, transmembrane and intracellular domains of CD28, and the intracellular domain of CD3ζ were designed in silico, cDNA was de novo synthesized, and subcloned into pSIEW or pSIRW lentiviral vectors (Fig. 1A). Then, ErbB2.CAR NK-92 cells were transduced with PD-L1.CAR and PD-L1.CAR2 constructs, resulting in dual PD-L1/ErbB2.CAR and PD-L1/ErbB2.CAR2 NK-92 cells (Fig. 1B). Simultaneously, parental NK-92 cells were transduced with PD-L1.CAR or PD-L1.CAR2, yielding PD-L1.CAR and PD-L1.CAR2 NK-92 cells. Both, PD-L1.CAR NK-92 and PD-L1/ErbB2.CAR NK-92 cells displayed comparable PD-L1.CAR expression (Fig. 1C, S1). Next, we tested functionality of the two PD-L1.CAR constructs using the ErbB2-negative breast cancer cell line MDA-MB-468 modified to ectopically overexpress the PD-L1 molecule (Fig. 1D). Thereby, PD-L1.CAR and PD-L1.CAR2 NK-92 cells lysed the PD-L1 positive target cells to a similar extent, but not the PD-L1 negative parental cells used as a control (Fig. 1E). Likewise, both dual PD-L1/ErbB2.CAR and PD-L1/ErbB2.CAR2 NK-92 cell lines specifically killed the PD-L1 positive target cells, demonstrating that co-expression with the ErbB2.CAR did not alter PD-L1.CAR functionality. Since PD-L1-directed activity and overall functional performance were largely comparable between PD-L1/ErbB2.CAR and PD-L1/ErbB2.CAR2 cells, with PD-L1/ErbB2.CAR showing a modest improvement in cytotoxic activity against PD-L1-positive targets, the majority of subsequent mechanistic and functional analyses were performed using PD-L1/ErbB2.CAR NK-92 cells, unless otherwise stated.

### Cytotoxicity of PD-L1/ErbB2 CAR NK-92 cells against solid tumor cells

**Fig. 2:**
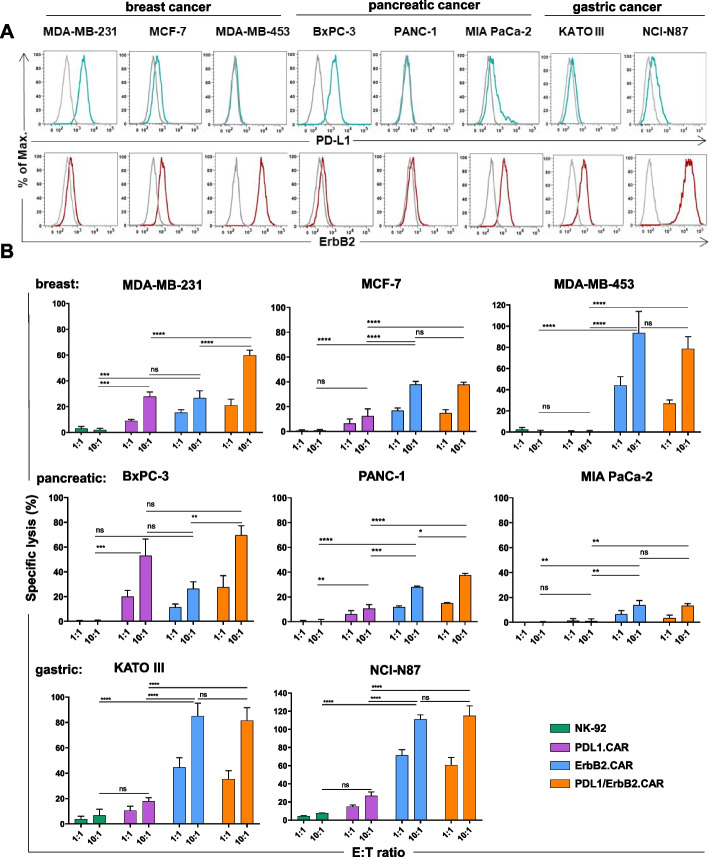
Dual PD-L1/ErbB2.CAR NK cells are cytotoxic against cancer cells from different solid tumors. **A** Established breast, pancreatic and gastric cancer cell lines were stained for cell surface expression of PD-L1 and ErbB2 and analyzed by flow cytometry as indicated. Gray lines indicate isotype controls. Representative data from at least 3 independent experiments are shown. **B** The indicated cancer cell lines were co-incubated with PD-L1/ErbB2.CAR, PD-L1.CAR, ErbB2.CAR or parental NK-92 cells at E:T ratios of 1:1 or 10:1 for 2 h, and specific lysis was measured using an Europium-based cytotoxicity assay. Data were pooled from at least n = 3 independent experiments. Mean values ± SEM are shown. ****, *p* < 0.0001; ***, *p* < 0.001; **, *p* < 0.01; ns (not significant), p ≥ 0.05

To analyze the cytotoxic activity of PD-L1/ErbB2.CAR NK-92 cells in more detail, we selected model tumor cell lines of different origin, including breast, pancreatic and gastric cancer. The cell lines expressed various levels of ErbB2 and PD-L1, simulating the natural heterogeneity of tumors (Fig. 2A). As expected, ErbB2.CAR NK-92 cells showed specific cytotoxicity against all ErbB2-positive cancer cells. Thereby, most pronounced cell killing activity was found against MDA-MB-453 breast carcinoma, and KATO III and NCI-N87 gastric cancer cells, which correlated with their high ErbB2 expression (Fig. 2B). PD-L1.CAR NK-92 cells specifically lysed cancer cells expressing PD-L1, with cell lines displaying higher PD-L1 levels, such as MDA-MB-231 breast carcinoma or BxPC-3 pancreatic cancer cells, killed to a larger extent than target cells with lower PD-L1 expression. In contrast, PD-L1 negative MDA-MB-453 cells were not lysed by PD-L1.CAR NK-92, confirming CAR-mediated selectivity of the effector cells. In the case of dual PD-L1/ErbB2.CAR NK-92 cells, cytotoxicity against the tested cancer cells reached at least the level of the most active single specificity CAR NK-92 cells. Importantly, enhanced cell killing activity of dual PD-L1/ErbB2.CAR NK-92 cells was found against MDA-MB-231, PANC-1 and BxPC-3 cells when compared to single CAR NK-92 controls. This effect was most pronounced for MDA-MB-231 target cells, where cell killing by the two single CAR NK-92 derivatives was similar (PD-L1.CAR 27.9 ± 6%; ErbB2.CAR 26.7 ± 9%), but dual CAR expression resulted in a more than additive effect on cytotoxicity (59.9 ± 6%). Interestingly, pancreatic MIA PaCa-2 cells were only killed to a very limited extent by the tested CAR-NK cells despite high ErbB2 and moderate PD-L1 expression, suggesting intrinsic resistance of the cancer cells against NK-cell cytotoxicity.

### Downstream signaling in PD-L1/ErbB2.CAR NK-92 cells

**Fig. 3:**
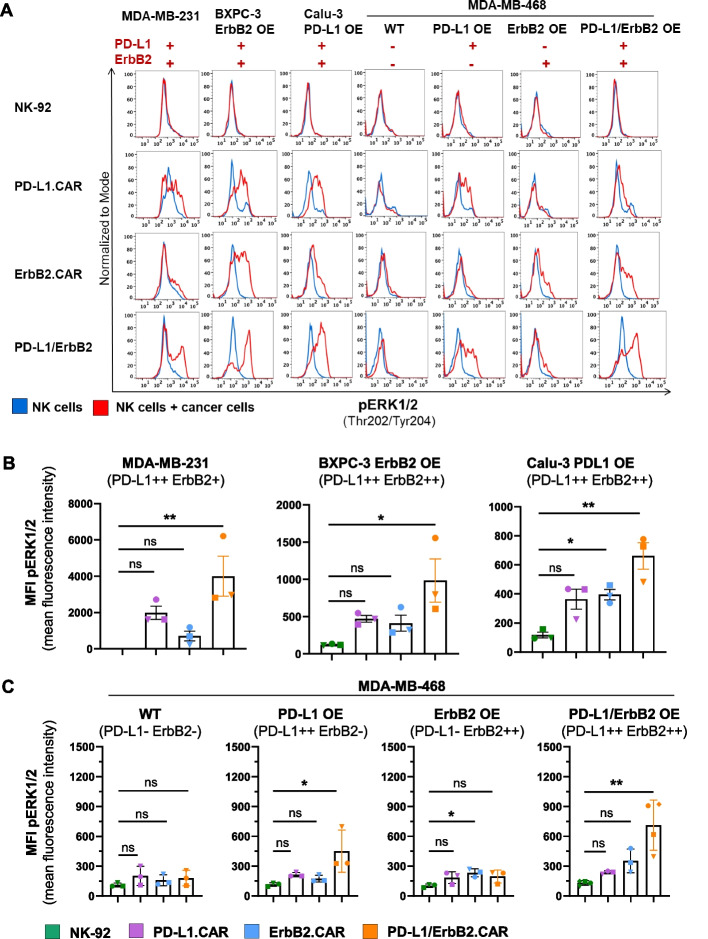
Cooperative downstream signaling in dual PD-L1/ErbB2.CAR NK-92 cells. PD-L1/ErbB2.CAR, PD-L1.CAR, ErbB2.CAR or parental NK-92 cells were co-cultured with PD-L1/ErbB2 double-positive MDA-MB-231 breast carcinoma, BXPC-3 ErbB2 OE pancreatic carcinoma, Calu-3 PDL1 OE lung carcinoma, double-negative MDA-MB-468 (WT), PD-L1-positive but ErbB2-negative MDA-MB-468 PD-L1 OE, PD-L1-negative but ErbB2-positive MDA-MB-468 ErbB2 OE, or double-positive MDA-MB-468 PD-L1/ErbB2 OE breast carcinoma cells for 60 min, or kept without target cells. Phosphorylation of the indicated signaling molecules in NK cells was determined by flow cytometry. Activation of the MEK/ERK pathway was analyzed by assessing pERK1/2 (Thr202/Tyr204). **A** Histograms from a representative experiment depicting NK cells in the presence (red) or absence (blue) of target cells. **B, C** Mean fluorescence intensity (MFI) data from n = 3 independent experiments. Mean values ± SEM are shown. One-way ANOVA was used for statistical analysis. **, *p* < 0.01; *, *p* < 0.05; ns (not significant), *p* ≥ 0.05

Next, we tested whether the enhanced cell killing activity of dual CAR NK-92 cells could be due to more potent activation of signaling pathways downstream of the CARs. For that, dual PD-L1/ErbB2.CAR, PD-L1.CAR, ErbB2.CAR and parental NK-92 cells were co-cultured with PD-L1 and ErbB2 double-positive MDA-MB-231 cells, followed by analysis of the phosphorylation of relevant signaling molecules in the MEK/ERK, PLCγ and PI3K pathways by flow cytometry. As expected, activation of single CARs induced increased phosphorylation of ERK when compared to unmodified NK-92 control cells (Fig. 3A, S2A, S3). This was further enhanced in dual CAR NK-92 cells. Likewise, more pronounced phosphorylation of PLCγ and Akt was found in activated PD-L1/ErbB2.CAR than in NK-92 cells expressing a single CAR or no CAR. While there was some inter-experimental variation, these results were consistent across 3 independent experiments (Fig. 3B). Similar data were obtained with endogenously PD-L1-expressing pancreatic (BXPC-3) and ErbB2-expressing lung (Calu-3) cancer cell lines modified to co-express ErbB2 (BXPC-3 ErbB2 OE) or PD-L1 (Calu-3 PDL1 OE) (Figure S2B) to model double-positive targets (Fig. 3A,B). In addition, we tested MDA-MB-468 breast cancer cell lines expressing none, either one, or both target antigens (Figure S2C), demonstrating that the observed CAR signaling was indeed antigen-dependent (Fig. 3C). To link signaling to functional outcomes, we performed degranulation assays. All CAR NK-92 cells exhibited a degranulation response upon stimulation with MDA-MB-231 cells (Figure S4A). Thereby, PD-L1/ErbB2.CAR NK-92 cells showed a trend toward more pronounced degranulation, although this did not reach statistical significance. To further assess the functional relevance of the identified signaling pathways, we performed pharmacological inhibition studies. Inhibition of the MEK1/2 pathway resulted in a moderate reduction of degranulation, suggesting partial redundancy (Figure S4B). In contrast, inhibition of PLCγ and PI3K/AKT pathways led to a pronounced decrease in degranulation of both single and dual CAR-NK cells, indicating their central role in mediating cytotoxic effector functions. Collectively, these data indicate that co-engagement of both target antigens enhances ERK, PLCγ, and AKT signaling in PD-L1/ErbB2 CAR-NK-92 cells more strongly than single-antigen stimulation, consistent with cooperative pathway activation triggering their increased cytotoxicity.

### Effect of IFN-γ on PD-L1 expression and sensitivity to PD-L1.CAR NK-92 cells

**Fig. 4:**
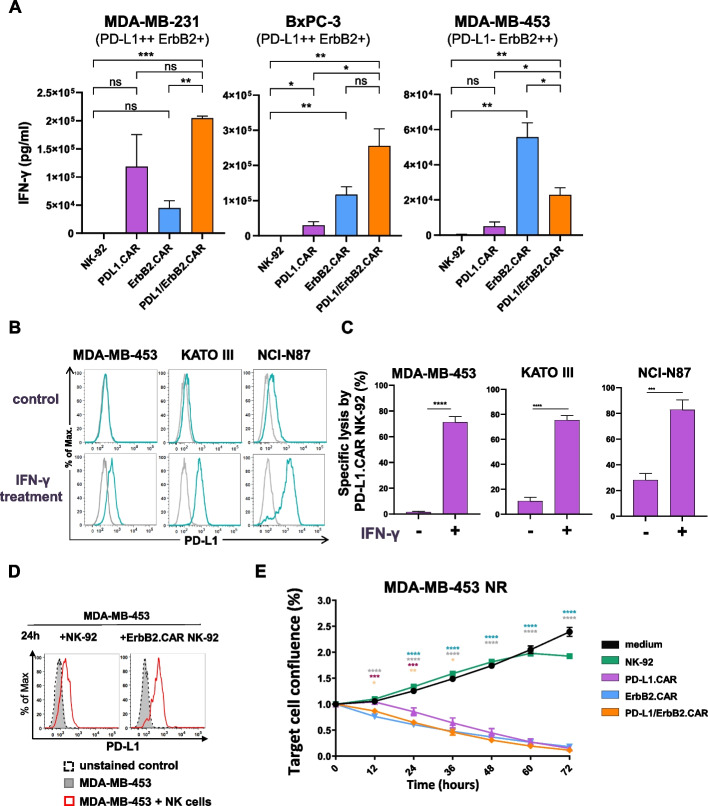
IFN-γ secreted by NK cells upregulates PD-L1 expression and sensitizes PD-L1 negative tumors to PD-L1.CAR targeting. **A** The indicated CAR-NK cells were co-cultured with MDA-MB-231, BxPC-3 or MDA-MB-453 cancer cells for 6 h at an E:T ratio of 1:1. Supernatants were collected, and the concentration of IFN-γ secreted by the effector cells was measured using a ProcartaPlex Luminex system. Data were pooled from n = 3 independent experiments, each performed in duplicates. Mean values ± SEM are shown. **B** The indicated cancer cells were incubated with 20 ng/mL of recombinant human IFN-γ (lower panels) or cultured without cytokines (upper panels) for 48 h. Then, upregulation of PD-L1 expression on the cell surface was analyzed by flow cytometry. Gray lines indicate unstained controls. **C** Cancer cells pretreated with IFN-γ or untreated controls were co-cultured with PD-L1.CAR NK-92 cells for 2 h at an E:T ratio of 10:1. Specific lysis was measured using an Europium-based cytotoxicity assay. **D** MDA-MB-453 cells were co-cultured with ErbB2.CAR or parental NK-92 cells for 24 h (red lines) or kept without effector cells (filled gray areas). Then, PD-L1 surface expression by the cancer cells was determined by flow cytometry. **E** MDA-MB-453 breast carcinoma cells expressing red fluorescent protein (Nuclight Red) were co-cultured with PD-L1/ErbB2.CAR, PD-L1.CAR, ErbB2.CAR or parental NK-92 cells at an E:T ratio of 1:1, and target cell confluency was monitored for 72 h using an IncuCyte live-cell imaging system. Data were pooled from n = 3 independent experiments. Statistical analysis was performed using two-way ANOVA. Mean values ± SEM are shown. ****, *p* < 0.0001; ***, *p* < 0.001; **, *p* < 0.01; *, *p* < 0.05; ns (not significant), p ≥ 0.05

An important characteristic of the antitumor activity of NK cells is the secretion of cytokines such as IFN-γ and TNF-α, as well as chemokines such as MIP-1α (CCL3) and MIP-1β (CCL4). To test cytokine production of PD-L1.CAR, ErbB2.CAR and PD-L1/ErbB2.CAR NK-92 cells upon encounter of target cells, the NK cells were co-cultured with PD-L1/ErbB2 double-positive MDA-MB-231 breast carcinoma and BxPC-3 pancreatic cancer cells, or ErbB2-positive but PD-L1-negative MDA-MB-453 breast carcinoma cells. Then, the amount of secreted cytokines into the supernatant by the NK cells was analyzed. Parental NK-92 cells were included as control. Consistent with the results of the cytotoxicity assays and signaling analysis, double-positive targets induced IFN-γ production by PD-L1/ErbB2.CAR NK-92 cells to the same or a higher extent when compared to single CAR NK-92 cells (Fig. 4A). Chemokines MIP-1α and MIP-1β were produced at robust levels by all CAR NK-92 cells, whereas TNF-α levels remained low across conditions (Figure S5). Conversely, ErbB2-positive MDA-MB-453 cells stimulated most effective secretion of IFN-γ in ErbB2.CAR NK-92 cells, without a significant effect on PD-L1.CAR or parental NK-92 cells (Fig. 4A). Interestingly, while still getting activated, dual-targeted PD-L1/ErbB2.CAR NK-92 secreted lower levels of IFN-γ than ErbB2.CAR cells in response to MDA-MB-453.

IFN-γ was previously shown to induce PD-L1 expression in cancer cells. Hence, presence of this cytokine could also upregulate PD-L1 on otherwise PD-L1-negative tumor cells in our model systems, making them accessible for targeting by the PD-L1.CAR. To test this hypothesis, we exposed PD-L1-negative/low breast (MDA-MB-453), gastric (KATO III, NCI-N87), and lung (A549, Calu-3) cancer cells to recombinant human IFN-γ, which upregulated endogenous PD-L1 expression, and in parallel generated PD-L1-overexpressing counterparts by lentiviral transduction (Fig. 4B, S6). Since these target cells express ErbB2 endogenously, we exposed them to PD-L1.CAR NK-92 cells to selectively test PD-L1.CAR-mediated cytotoxicity independent of the ErbB2.CAR. As expected, PD-L1.CAR NK-92 cells failed to kill the untreated PD-L1 negative/low cancer cells in short-term (2 h) killing assays. However, marked PD-L1.CAR-mediated cytotoxicity was found after IFN-γ- or genetically induced PD-L1 upregulation (Fig. 4C, S7, S8). The same effect was observed with PD-L1.CAR2 NK-92 cells (Figure S6).

Next, we tested whether prolonged incubation of PD-L1-negative cancer cells with NK cells could lead to PD-L1 upregulation induced by the IFN-γ naturally secreted by the NK cells, potentially facilitating subsequent killing by PD-L1.CAR NK-92. First, PD-L1-negative but ErbB2-positive MDA-MB-453 cells were co-cultured with ErbB2.CAR or parental NK-92 cells. Thereby, exposure to ErbB2.CAR NK-92 cells and soluble factors secreted by the NK cells led to a strong upregulation of PD-L1 on the surface of MDA-MB-453 cells. To some extent this was also the case with parental NK-92 cells (Fig. 4D). Likewise, in a transwell assay with ErbB2-positive A549 lung cancer cells, PD-L1 upregulation was found after exposure to ErbB2.CAR NK-92 cells (Figure S10). To directly assess the role of IFN-γ, we performed neutralization experiments. Co-culture of MDA-MB-453 cells with ErbB2.CAR NK-92 cells in the presence of neutralizing antibodies revealed that blockade of IFN-γ significantly reduced PD-L1 upregulation, whereas TNF-α blockade alone had no significant effect (Figure S9). Combined inhibition of IFN-γ and TNF-α resulted in a more pronounced reduction in PD-L1 upregulation. This indicates a minor cooperative, but not independent, contribution of TNF-α, while IFN-γ appears to be the primary driver of PD-L1 induction.

Next, we co-cultured MDA-MB-453 cells with PD-L1/ErbB2.CAR, ErbB2.CAR, PD-L1.CAR or parental NK-92 cells for three days, and continuously monitored cytotoxicity using a real-time live cell imaging system. As expected both, ErbB2.CAR and PD-L1/ErbB2.CAR NK-92 cells killed the ErbB2-positive targets, whereas unmodified NK-92 did not (Fig. 4E, S11). Importantly, while PD-L1.CAR NK-92 cells had no effect on the viability of MDA-MB-453 cells during the first 12 h of co-culture, marked cytotoxicity was observed at later time points, and reached levels comparable to those of ErbB2.CAR and PD-L1/ErbB2.CAR NK-92 cells at 72 h. Taken together, these data demonstrate that PD-L1 expression on initially PD-L1-negative cancer cells can indeed be stimulated by IFN-γ produced by the NK cells, thereby rendering them susceptible to targeting by PD-L1.CAR-expressing effector cells.

### Activity of dual CAR-NK cells against three-dimensional tumor spheres

**Fig. 5:**
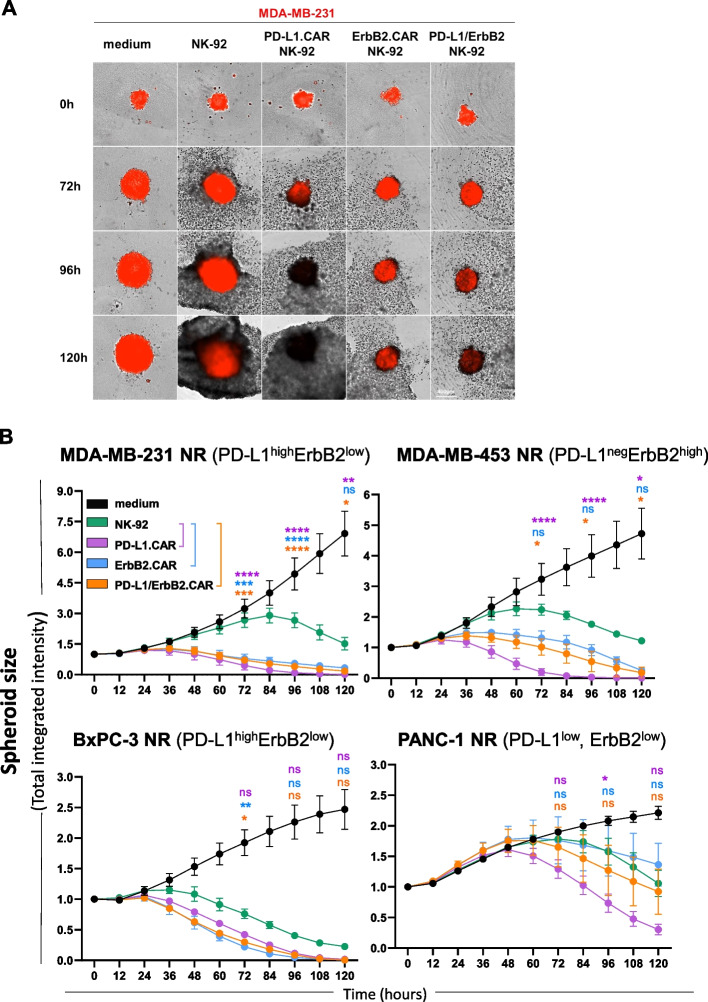
Dual PD-L1/ErbB2.CAR-NK cells eliminate cancer cells growing as 3D spheroids. **A** MDA-MB-231 breast carcinoma cells expressing red fluorescent protein (Nuclight Red) were mixed with Matrigel, and cultured in low-adhesion plates for 24 h to allow formation of tumor spheroids. Then, the indicated CAR-NK cells were added at an E:T ratio of 1:1, and spheroid size was monitored every 12 h for a total of 5 days using an IncuCyte live-cell imaging system. Parental NK-92 cells were included for comparison. Samples without effector cells served as control. Representative images are shown. **B** Quantification of spheroid size over time, measured as total integrated fluorescence intensity, for MDA-MB-231, MDA-MB-453, BxPC-3, and PANC-1 spheroids. n = 3 independent experiments. Mean values ± SEM are shown. Statistical analysis was performed using two-way ANOVA (indicated for 72, 96 and 120 h time points). ****, *p* < 0.0001; ***, *p* < 0.001; **, *p* < 0.01; *, *p* < 0.05; ns (not significant), *p* ≥ 0.05

One of the challenges for cellular therapy of solid tumors is their three-dimensional structure, which can impede immune cell infiltration and create an unfavorable inhibitory environment. To test the antitumoral activity of PD-L1/ErbB2.CAR NK-92 cells in 3D in vitro systems more similar to tumor tissues than regular cell cultures, we established spheroid models of breast (MDA-MB-231, MDA-MB-453) and pancreatic (BxPC-3, PANC-1) cancer cells. CAR-engineered NK-92 cells were added to spheroids labeled with a fluorescent dye, and cell killing activity was analyzed using a live-cell imaging system. Over the course of the five day experiments, PD-L1/ErbB2.CAR NK-92 cells lysed PD-L1^high^ErbB2^low^ MDA-MB-231 and BxPC-3 spheroids to a similar extent as single CAR NK-92 cells, and more effectively than unmodified NK-92. PD-L1^neg^ErbB2^high^ MDA-MB-453 spheroids were efficiently killed by ErbB2.CAR NK-92 and PD-L1/ErbB2.CAR NK-92 cells, with parental NK-92 cells again showing only limited activity (Fig. 5A,B, S12). Surprisingly, PD-L1.CAR NK-92 cells outperformed both, PD-L1/ErbB2.CAR NK-92 and ErbB2.CAR NK-92 cells against MDA-MB-453 and PD-L1^neg/low^ErbB2^low^ PANC-1 spheroids. This suggests that similar to the 2D culture experiments described above, prolonged exposure to soluble factors released by the NK cells had induced PD-L1 upregulation. Collectively, these data demonstrate potent activity of ErbB2.CAR and PD-L1.CAR as well as dual PD-L1/ErbB2.CAR NK-92 cells against three-dimensional tumor cell structures.

### Overcoming immune escape induced due to antigen heterogeneity

**Fig. 6:**
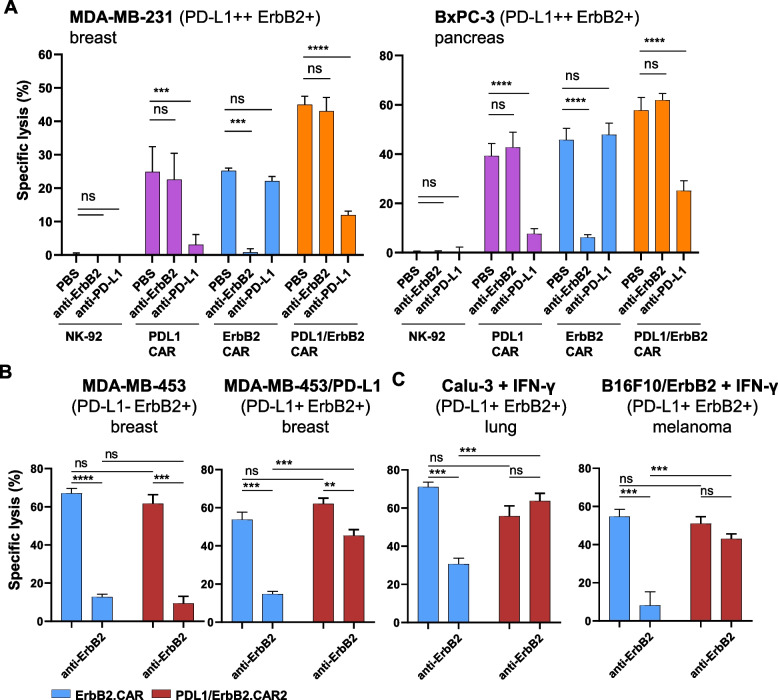
Loss of ErbB2 or PD-L1 antigens can be overcome by dual PD-L1/ErbB2.CAR-NK cells. **A** ErbB2 and PD-L1 target antigens on MDA-MB-231 breast carcinoma and BxPC-3 pancreatic cancer cells were blocked with specific antibodies against ErbB2, PD-L1 or both and the cells were co-cultured with dual PD-L1/ErbB2.CAR, PD-L1.CAR, ErbB2.CAR or parental NK-92 for 2 h. Specific cytotoxicity was then measured using an Europium-based assay. Mean values ± SEM are shown; n = 3 independent experiments. In similar assays, ErbB2-positive but PD-L1-negative MDA-MB-453 and PD-L1-overexpressing MDA-MB-453/PD-L1 breast carcinoma cells (**B**), or IFN-γ-treated Calu-3 lung cancer and B16F10/ErbB2 melanoma cells (**C**) were treated with a blocking anti-ErbB2 antibody and then co-cultured with PD-L1/ErbB2.CAR2 or ErbB2.CAR NK-92 cells for 2 h at an E:T ratio of 10:1. Specific cytotoxicity was determined using a flow cytometry-based cytotoxicity assay. Mean values ± SD are shown; n = 3 biological replicates. ****, *p* < 0.0001; ***, *p* < 0.001; **, *p* < 0.01; ns (not significant), *p* ≥ 0.05

Antigen loss can lead to treatment failure with targeted therapeutics, and is a common problem for CAR-based therapies. To examine whether dual targeting could address this issue, we mimicked antigen loss by blocking the ErbB2 or PD-L1 antigens on ErbB2/PD-L1 double-positive MDA-MB-231 and BxPC-3 cancer cells with blocking antibodies, and co-cultured them with CAR-engineered NK cells. As expected, ErbB2.CAR NK-92 cells failed to kill the targets when ErbB2 was blocked. Likewise, the activity of PD-L1.CAR NK-92 cells was inhibited in the presence of a PD-L1-blocking antibody. The anti-PD-L1 antibody did not affect activity of ErbB2.CAR NK-92, and the anti-ErbB2 antibody did not inhibit PD-L1.CAR NK-92 cells, confirming specificity of the observed effects (Fig. 6A). In contrast, PD-L1/ErbB2.CAR NK-92 cells still exhibited marked cell killing activity if either ErbB2 or PD-L1 were blocked. Thereby, cytotoxicity of the dual CAR-NK cells remained largely unaffected by the anti-ErbB2 antibody, while blockade of PD-L1 reduced cytotoxicity to some extent, suggesting that the PD-L1.CAR played a more dominant role in this setting, with tumor cells expressing higher levels of PD-L1 than ErbB2 on their surface. We also investigated the effects of the second PD-L1/ErbB2.CAR2 NK-92 cell line against ErbB2-positive but initially PD-L1-negative tumor cells upon blockade of ErbB2. Since the PD-L1.CAR2 is derived from atezolizumab, like the original antibody the respective CAR can interact with both, human and murine PD-L1. As expected, in short-term killing assays blockade of ErbB2 in PD-L1-negative MDA-MB-453 cells abrogated cytotoxicity of both, ErbB2.CAR and PD-L1/ErbB2.CAR2 NK-92 cells (Fig. 6B). However, ectopic overexpression of PD-L1 fully restored cell killing by PD-L1/ErbB2.CAR2 NK-92 cells even in the presence of the ErbB2-blocking antibody. Similarly, despite blockade of ErbB2, ErbB2-positive but initially PD-L1-negative human lung (Calu-3) and mouse melanoma (B16F10/ErbB2) cells were readily killed by the PD-L1/ErbB2.CAR2 cells if endogenous PD-L1 expression was upregulated by treatment with human or murine IFN-γ (Fig. 6C). Taken together, these data demonstrate that, unlike single-specificity effectors, dual ErbB2/PD-L1 CAR-NK-92 cells can overcome single-antigen heterogeneity by retaining cytotoxic function as long as either ErbB2 or PD-L1 is present or rendered targetable through IFN-γ mediated induction.

### Assessment of potential off-tumor effects and fratricide

To evaluate potential fratricide, PD-L1 expression was assessed on all CAR-NK-92 variants. No detectable PD-L1 expression was observed, and no reduction in viability was detected across CAR-NK-92 cells, indicating absence of fratricide (Figure S13). To assess potential off-tumor cytotoxicity, co-culture experiments with primary human PBMCs and mesenchymal stem cells (MSCs) were performed. Both PBMCs and MSCs displayed no detectable PD-L1 or ErbB2 expression. Still, low-level PD-L1 expression induced by isolation or culture stress, below the detection limit of flow cytometry, cannot be excluded. Dual PD-L1/ErbB2.CAR NK-92 cells exhibited only limited cytotoxicity toward PBMCs and MSCs, even at a high E:T ratio of 10:1 (Figure S14). These findings suggest that cytotoxic activity toward non-malignant cells is limited under baseline conditions with low target antigen expression.

### Dual PD-L1/ErbB2.CAR expression and function in primary NK cells

To explore the broader applicability of the dual CAR strategy beyond NK-92 cells, we performed initial experiments using primary peripheral blood-derived NK cells from healthy donors. Although co-transduction with the CAR vectors was feasible and antigen-specific cytotoxicity of the transduced NK cells against PD-L1/ErbB2-positive target cells was confirmed (Figure S15), the frequency of CAR-positive cells was lower and more variable compared to NK-92 cells, which allow the generation of homogeneous and well-defined dual CAR-positive populations. These findings demonstrate the feasibility of dual PD-L1/ErbB2 CAR expression in primary NK cells; however, further optimization of transduction efficacy will be required for robust application in primary NK cell systems.

### Cytotoxicity of dual PD-L1/ErbB2.CAR NK-92 cells against patient-derived primary ovarian cancer cells

**Fig. 7:**
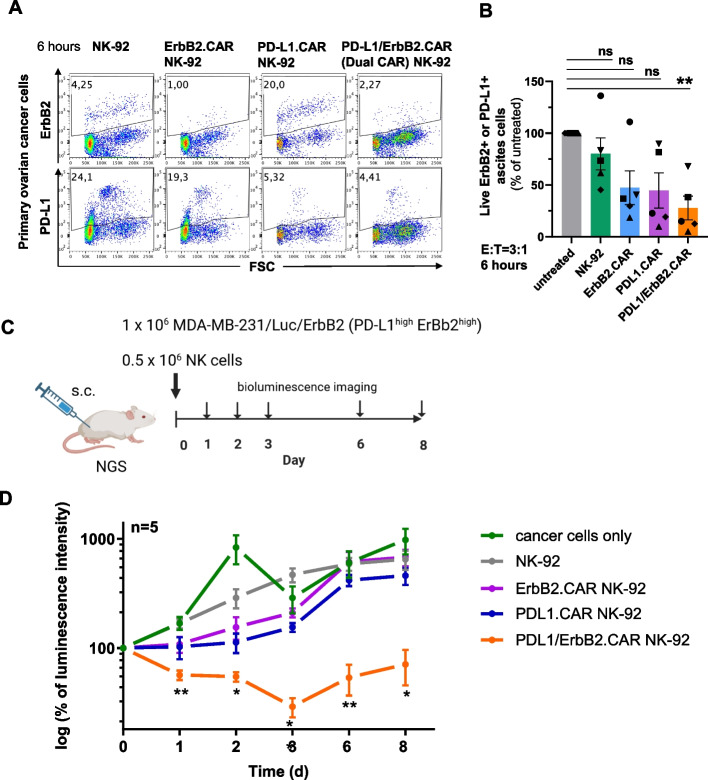
Dual PD-L1/ErbB2.CAR-NK cells show efficacy against primary ovarian cancer cells and in a preclinical breast cancer xenograft model. **A** Ascites cells isolated from a primary ovarian cancer patients were cultured with NK cells for 6 h. The frequencies of live, NK cell-excluded ErbB2⁺ and PD-L1⁺ cells were analyzed by flow cytometry. **B** Frequency of ErbB2⁺ or PD-L1⁺ cells in ascites cultured alone or co-cultured at E:T ratio 3:1 with NK-92 cells or CAR-NK cells, as indicated. Mean values ± SEM are shown; n = 5 patients. (**C**) In vivo activity of CAR-NK cells was investigated in a breast carcinoma xenograft model with PD-L1-positive MDA-MB-231/Luc/ErbB2 cells modified to express firefly luciferase and enhanced levels of ErbB2. 1 × 10^6^ cancer cells were mixed with 0.5 × 10.^6^ PD-L1/ErbB2.CAR, PD-L1.CAR, ErbB2.CAR or parental NK-92 cells, and injected subcutaneously into female NXG mice (*n* = 5). **D** Tumor growth was followed by bioluminescence imaging for 8 days as indicated. Data are represented as mean values ± SD. Statistical analysis was performed using two-way ANOVA and Dunnett multiple-comparison test, and is indicated for differences between the PD-L1/ErbB2.CAR-treated group and animals injected with parental NK-92 cells. ****, *p* < 0.0001, **, *p* < 0.01; *, *p* < 0.05, ns (not significant), *p* ≥ 0.05

We next employed a patient-derived ovarian cancer model, NK-resistant primary ovarian cancer ascites isolated from malignant ascites of five FIGO stage III–IV patients, containing heterogeneous cancer cell populations that expressed varying levels of ErbB2 and PD-L1 using tumor cells (Fig. 7A). The ascites-derived cells were co-cultured with CAR-NK-92 cells for 6 h and analyzed by flow cytometry for the frequency of ErbB2⁺ and PD-L1⁺ cancer cells. The presence of parental NK-92 cells resulted only in a marginal decline of cancer cells in samples from some patients, whereas single CAR-NK-92 cells showed increased specific cytotoxicity against target antigen-expressing cancer cells (Fig. 7B, S16, S17). Of note, dual PD-L1/ErbB2.CAR NK-92 cells displayed further enhanced cytotoxicity against the majority of the patient-derived cancer cells. Thereby, the largest benefit of the dual CAR system was observed with primary tumor samples containing heterogeneous population of both ErbB2- and PD-L1-positive cells. While single CAR-NK-92 cells only eliminated cancer cells expressing the respective single target antigen, dual CAR-NK-92 cells were able to eliminate both populations (Fig. 7A). To further validate these findings using an independent assay, we next assessed the cytotoxic activity of PD-L1/ErbB2.CAR NK-92 cells against primary ovarian cancer cells in a direct cytotoxicity assay. Due to the limited availability of primary tumor material, PD-L1.CAR NK-92 cells could not be included in this analysis. Freshly isolated ovarian cancer cells were co-cultured with dual PD-L1/ErbB2.CAR NK-92, ErbB2.CAR NK-92 or parental NK-92 cells. Under these conditions, the primary tumor cells were largely resistant to unmodified NK-92 cells (Figure S18). Consistent with their low ErbB2 expression, only limited cytotoxicity was observed with ErbB2.CAR NK-92 cells. In contrast, dual PD-L1/ErbB2.CAR NK-92 cells exhibited markedly enhanced cytotoxicity and efficiently eliminated ErbB2^low^PD-L1^int^ primary ovarian cancer cells.

### In vivo efficacy of dual PD-L1/ErbB2.CAR NK-92 cells

To test antitumoral activity of the PD-L1/ErbB2.CAR NK-92 cells in vivo, we established a mouse xenograft model based on MDA-MB-231 breast carcinoma cells modified to overexpress ErbB2 and a luciferase reporter gene. The resulting ErbB2^high^PD-L1^high^ MDA-MB-231/Luc/ErbB2 cells were subcutaneously injected in immunodeficient NXG mice together with PD-L1/ErbB2.CAR NK-92 cells or controls at a low E:T ratio of 1:2. Tumor growth over time was followed with bioluminescence imaging (Fig. 7C). While the observed differences were not statistically significant, treatment with both, ErbB2.CAR and PD-L1.CAR NK-92 cells had some effect, leading to moderate but transient inhibition of in vivo growth of the PD-L1/ErbB2 double-positive cancer cells when compared to unmodified NK-92 cells or untreated controls (Fig. 7D, S19), consistent with the low E:T conditions in this model. Conversely, PD-L1/ErbB2.CAR NK-92 cells demonstrated superior in vivo activity, resulting in a significant reduction of tumor size over several days after a single injection of the NK cells.

Together, these results demonstrate that dual PD-L1/ErbB2 CAR-NK-92 cells exhibit potent cytotoxicity against patient-derived primary ovarian cancer cells, and mediate superior in vivo tumor control in a xenograft model of PD-L1/ErbB2-positive breast cancer.

## Discussion

Treatment with CAR-T cells has led to remarkable clinical responses in hematological malignancies, and early clinical data with CD19-specific CAR-NK cells derived from allogeneic donor cells demonstrated comparable therapeutic activity without the side effects typically observed after CAR-T cell application [12, 13]. Nevertheless, more prevalent solid tumors like breast, ovarian, lung and pancreatic cancers still remain a challenge for CAR-based therapies. Limited infiltration into the tumor tissue, an immunosuppressive tumor microenvironment, the presence of T-cell inhibitory immune checkpoint molecules, and heterogeneous expression of CAR target antigens represent major obstacles for adoptively transferred effector lymphocytes [48, 49].

Addressing some of these challenges, here we generated dual PD-L1/ErbB2.CAR-NK cells based on the clinically applied ErbB2.CAR NK-92 cell line NK-92/5.28.z [24]. The CAR effector cells with dual specificity allowed targeting of more than one antigen, and resulted in efficient lysis of cancer cells that express the cellular proto-oncogene ErbB2, the PD-L1 immune checkpoint molecule, or both antigens. Thereby, specific killing of PD-L1-positive cells in the tumor can be expected to also eliminate immune inhibitory signals, re-invigorate the activity of endogenous T cells present in the vicinity, and further enhance the immunomodulatory effects of the CAR-engineered NK-92 cells previously observed in immunocompetent animal models and cancer patients [24, 29, 50]. Employing two different PD-L1-specific scFv antibody fragments for CAR construction [21, 42], our study demonstrated efficient cytotoxicity of both types of dual PD-L1/ErbB2.CAR-NK cells generated (PD-L1/ErbB2.CAR and PD-L1/ErbB2.CAR2 NK-92) against a wide variety of cancer cells derived from solid tumors like breast, ovarian, pancreatic, gastric and lung cancers. Importantly, high anti-tumoral activity was retained against patient-derived primary cancer cells and cancer cells growing as spheroids, with the latter indicating that the dual CAR-NK cells can also penetrate and eliminate three-dimensional structures, which provide a better in vitro model of a tumor tissue than regular cell cultures. A single treatment with the dual PD-L1/ErbB2.CAR NK-92 cells at a low effector to target cell ratio also significantly delayed tumor outgrowth in a subcutaneous breast cancer xenograft model in immunodeficient mice, which was not observed with single-specificity PD-L1.CAR and ErbB2.CAR NK-92. These in vivo data provide initial support for enhanced antitumor activity of dual PD-L1/ErbB2.CAR NK-92 cells. Still, the simplified xenograft model used in this study has limitations. Hence, also extensive testing in human-relevant experimental systems, including multiple cancer cell lines with heterogeneous PD-L1 and ErbB2 expression, advanced 3D tumor spheroid models, and patient-derived primary ovarian cancer cells was performed, which all convincingly demonstrated superior activity of the dual CAR-NK cells. Nevertheless, future studies using additional complementary preclinical models and, ultimately, careful clinical investigation will be required to fully assess long-term efficacy of PD-L1/ErbB2.CAR NK-92 cells, persistence, and tumor evolution under therapeutic pressure. Thereby, a novel metastatic cancer xenograft model using zebrafish larvae may be useful, which we recently introduced for testing of CAR-NK cells [40]. This model may allow to evaluate in a future study the anti-metastatic potential of dual PD-L1/ErbB2.CAR NK-92 cells with patient-derived cancer cells, and identify tumor entities and patient cohorts that may benefit most from such treatment.

Importantly, even for cancer cells that expressed both, PD-L1 and ErbB2, and were partially sensitive to single-specificity PD-L1.CAR or ErbB2.CAR NK-92 cells, we observed improved cytotoxicity of the dual CAR-NK cells. This was also evident in the enhanced activation of the ERK, PLCγ and PI3K signaling pathways following contact of the NK cells with PD-L1 and ErbB2 double-positive cancer cells. Notably, all CAR molecules used in our study employ the same composite CD28-CD3ζ signaling domain previously shown to be highly effective in CAR-engineered NK-92 and primary NK cells [28, 51, 52]. Nevertheless, our approach also opens the possibility of instead using different signaling units with complementary activity or different downstream targets, enabling alternative logic-gated strategies [53, 54]. This could allow further amplification or more fine-tuned modulation of signals based on the antigen expression profile of the targeted cancer cells. While the PD-L1/ErbB2.CAR NK-92 cells were effective against all other established cancer cell lines and primary cancer cells tested, pancreatic MIA PaCa-2 cells proved highly resistant to CAR-mediated cytotoxicity, despite expression of the corresponding antigens. This is indicative of resistance mechanisms unrelated to a lack of the CAR target antigens, and merits further investigation. A better understanding of such mechanism will likely be instructive to rationally improve the design of NK-cell based therapeutics.

We and others have shown that PD-L1.CAR-based killing is dependent on the extent of PD-L1 expression by the target cells [21, 36]. While many tumors are PD-L1-positive, PD-L1 levels can be relatively low, and not all cancer cells within a given tumor may homogeneously express the antigen [37]. Hence, a variable proportion of the cancer cells cannot be targeted by PD-L1-specific CAR-NK cells. Nevertheless, in our study exposure to recombinant IFN-γ upregulated PD-L1 expression, resulting in enhanced cell killing by PD-L1.CAR NK-92 cells, which is consistent with previous reports [21, 34]. Furthermore, we demonstrated that also soluble factors secreted by activated ErbB2.CAR and PD-L1/ErbB2.CAR NK-92 cells can induce PD-L1 upregulation in co-cultured cancer cells, primarily due to the high amounts of IFN-γ secreted by the effector cells. Consequently, in long-term cytotoxicity experiments with ErbB2-positive but initially PD-L1-negative or PD-L1^low^ cancer cells, this induced upregulation of PD-L1 rendered the target cells eventually also susceptible to PD-L1.CAR-mediated cytotoxicity. Expanded cytokine profiling further supports this mechanism, revealing also robust secretion of MIP-1α and MIP-1β, while TNF-α levels remained low upon CAR-NK cell activation. Blockade of IFN-γ significantly reduced PD-L1 upregulation, whereas TNF-α inhibition had no measurable effect, indicating IFN-γ as the dominant driver of PD-L1 induction. Together, these findings support a self-amplifying feedback mechanism in which CAR-NK-derived IFN-γ enhances PD-L1 expression and increases tumor susceptibility to PD-L1-targeted cytotoxicity.

The enhanced phosphorylation of ERK, AKT, and PLCγ observed in dual CAR-NK cells is likely a consequence of simultaneous engagement of two independent CARs sharing identical intracellular signaling domains. Co-recognition of PD-L1 and ErbB2 may increase the number and duration of activation events at the immune synapse, resulting in cumulative downstream signaling. Functionally, this translates into enhanced NK cell activation, as reflected by increased degranulation and a robust cytokine secretion profile. This is consistent with our previous studies dissecting CAR-NK signaling pathways, as well as with mechanistic studies demonstrating that lytic granule polarization and subsequent degranulation are critical for CAR-mediated cytotoxicity [30, 31]. Pharmacological inhibition studies further indicated that PLCγ and PI3K/AKT signaling play a central and less redundant role in mediating cytotoxic effector functions, whereas MEK/ERK signaling appears to contribute in a more modulatory manner.

The limited cytotoxicity observed toward primary non-malignant cells, together with the absence of fratricide, suggests a manageable safety profile in vitro. However, as PD-L1 is not tumor-restricted and can be expressed on healthy tissues or induced under inflammatory conditions, potential on-target/off-tumor effects cannot be fully excluded at this point. Careful clinical translation, including step-wise dose escalation and close monitoring, will be essential to assess safety in patients.

Using different preclinical cancer models, we showed that dual PD-L1/ErbB2.CAR NK-92 cells are not affected by the absence or blockade of either one of the target antigens, suggesting that they could circumvent immune escape due to antigen loss or heterogeneous expression of PD-L1 and ErbB2 within a tumor. Moreover, our results indicate that the two CARs operate independently, and do not require both antigens to be present for efficient NK-cell activation. In a previous study with glioblastoma cells that express epidermal growth factor receptor (EGFR) and its tumor-specific variant EGFRvIII, therapy with CAR-NK cells targeting either EGFR or EGFRvIII resulted in treatment-induced outgrowth of escape variants, while a single CAR with an antibody domain recognizing an epitope common to both target antigens was still effective [55]. Likewise, tandem CARs combining two different binding domains in a single CAR construct circumvent immune escape [35]. Such a combination of two binders in the same CAR molecule could also be applied to target ErbB2 and PD-L1. Nevertheless, synergistic signaling arising from two distinct CARs may still be more beneficial and result in further enhanced cytotoxicity against cancer cells expressing both target antigens. A dual CAR strategy using CD19- and CD20-specific binders expressed on distinct CAR molecules but sharing identical intracellular signaling domains, including a 4-1BB costimulatory domain, has previously been described for targeting hematological malignancies [56]. However, cooperative CAR signaling was not investigated in that study. Future studies will therefore be required to determine whether the presence of the 4-1BB costimulatory domain can mediate cooperative or synergistic signaling between two independently expressed CARs. In another study, dual-CAR systems combining an activating CAR with a second inhibitory CAR have been described [57], an approach that could potentially be integrated with our strategy in the future to mitigate trogocytosis-mediated fratricide as well as the risk of on-target, off-tumor toxicities.

We tested two distinct PD-L1.CARs derived from different PD-L1-specific antibodies for co-expression with the ErbB2.CAR [21, 42]. Both showed comparable functionality against multiple targets, suggesting that neither one interacted with the co-expressed ErbB2.CAR in a manner negatively affecting CAR activity. This may be different if two binding domains are combined in a tandem CAR, where positioning of the scFv fragments and connecting linker sequences could have a decisive impact [35]. Alternatively, a mixture of individual PD-L1.CAR and ErbB2.CAR-NK cells could be employed for dual targeting, as has been previously shown for CAR-T cells [34, 35]. While such an approach would allow personalized dosing by selecting a patient-individual ratio of the CAR effector cells, similar to a tandem CAR, possible synergistic signaling effects of both CARs would be lost. Furthermore, this would require to manufacture two separate clinical products, which may be feasible and cost-effective for allogeneic off-the-shelf therapeutics like NK-92, but most likely not for cell products derived from patients or healthy donors.

While our results demonstrate the therapeutic potential of dual PD-L1/ErbB2.CAR NK-92 cells, important restrictions of the NK-92 platform must be considered when interpreting their translational applicability. It is current clinical practice that NK-92 cells are irradiated prior to infusion, which limits their in vivo persistence and proliferative capacity. Consequently, sustained therapeutic efficacy will likely depend on repeated dosing strategies and/or the activation of endogenous cellular and humoral immune responses, rather than long-term engraftment of the infused cells.

## Conclusion

Taken together, our data demonstrate markedly improved antitumor activity of dual PD-L1/ErbB2.CAR-NK cells when compared to single-specificity PD-L1.CAR and ErbB2.CAR NK-92 against PD-L1-, ErbB2- or double-positive cancer cells from different solid tumor entities. Cytotoxic activity was retained when one of the target antigens was not present or became inaccessible, indicating that PD-L1/ErbB2.CAR-NK cells can circumvent immune escape due to antigen loss. Since PD-L1 is predominantly expressed by cancer cells and immunosuppressive myeloid cell subtypes, it constitutes an ideal second target for dual-targeting strategies with respect to potential on-target/off-tumor toxicities. Furthermore, we based the PD-L1/ErbB2.CAR-NK cells on the ErbB2.CAR cell line NK-92/5.28.z, which already demonstrated safety as an off-the-shelf therapeutic in cancer patients. This is expected to aid further development, although additional preclinical studies are required to assess the therapeutic potential of PD-L1/ErbB2.CAR-NK cell products in more detail before initiating clinical translation.

## Supplementary Information

Supplementary Material 1: Figure S1: (A) CAR expression on PD-L1/ErbB2.CAR, PD-L1.CAR and ErbB2.CAR NK-92 cells was determined by flow cytometry using recombinant ErbB2-Fc or PD-L1-Fc protein, followed by staining with an anti-Fc secondary antibody. Parental NK-92 cells were included for comparison. (B) CAR expression on PD-L1/ErbB2.CAR2, PD-L1.CAR2 and ErbB2.CAR NK-92 cells was determined by flow cytometry. Filled gray areas indicate negative controls only stained with secondary antibody. Representative data from at least 3 independent experiments are shown. Figure S2: (A) Gating strategy applied for the flow cytometric protein phosphorylation analysis shown in Figures 3 and S3. Shown are representative data upon co-culture of NK cells with MDA-MB-231 cancer cells. Singlets were selected based on FSC and SSC parameters. NK cells were identified by CD56 staining, and further analyzed with anti-phosphoprotein antibodies as depicted in Figures 3 A and S3. (B,C) Flow cytometry data histograms showing expression of PD-L1 (blue) and ErbB2 (red) by BXPC-3 ErbB2 OE (ErbB2 overexpressed), Calu-3 PDL1 OE (PD-L1 overexpressed), MDA-MB-468 (WT), MDA-MB-468 PD-L1 OE (PD-L1 overexpressed), MDA-MB-468 ErbB2 OE (ErbB2 overexpressed), and MDA-MB-468 PD-L1/ErbB2 OE (PD-L1 and ErbB2 overexpressed) cells. Figure S3: Downstream PLCγ and PI3K signaling in dual PD-L1/ErbB2.CAR NK-92 cells. PD-L1/ErbB2.CAR, PD-L1.CAR, ErbB2.CAR or parental NK-92 cells were co-cultured with PD-1/ErbB2 double-positive MDA-MB-231 breast carcinoma cells for 20 or 60 min, or kept without target cells. Phosphorylation of the indicated signaling molecules in NK cells was determined by flow cytometry. Activation of PLCγ and PI3K pathways was analyzed by assessing pPLCγ1 (Ser1248) and pAkt (Ser473), respectively. (A) Histograms from a representative experiment depicting NK cells in the presence (red) or absence (blue) of target cells. (B) Mean fluorescence intensity (MFI) data from n=3 independent experiments. Mean values ± SEM are shown. One-way ANOVA was used for statistical analysis. *, p<0.05; ns (not significant), p≥0.05. Figure S4: (A) PD-L1/ErbB2.CAR NK-92 cells and corresponding control cells were co-cultured with Nuclight Red expressing MDA-MB-231 NR cells for 90 minutes at an E:T ratio of 1:1 in the presence of anti-CD107a antibody. The frequency of CD107a-positive NK cells was determined by flow cytometry. NK-92 cells were identified as CD56⁺ and Nuclight Red-negative. One-way ANOVA was used for statistical analysis. (B) CAR NK-92 and parental NK-92 cells were pre-incubated with inhibitors targeting MEK1/2, PLCγ, or PI3K, or with mock control, for 60 minutes as indicated, followed by co-culture with MDA-MB-231 cells for 90 minutes at an E:T ratio of 1:1 in the presence of anti-CD107a antibody. Data were analyzed by flow cytometry, and the frequency of CD107a-positive cells is shown. Data represent pooled results from three independent experiments. Two-way ANOVA was used for statistical analysis. Mean values ± SEM are shown. ****, p<0.0001; ***, p<0.001; **, p<0.01; *, p<0.05; ns (not significant) p≥0.05. Figure S5: The indicated CAR-NK cells were co-cultured with MDA-MB-231 breast cancer cells for 6 hours at an E:T ratio of 1:1. Supernatants were collected, and the concentration of TNF-α, MIP-1α (CCL3), and MIP-1β (CCL4) secreted by the effector cells was measured using a ProcartaPlex Luminex system. Data were pooled from n=3 independent experiments, each performed in duplicates. Mean values ± SEM are shown. One-way ANOVA was used for statistical analysis. ****, p<0.0001; ns (not significant) p≥0.05. Figure S6: PD-L1 expression upon IFN-γ treatment or transduction with a PD-L1 encoding vector. (A) Human A549 and Calu-3 lung cancer and murine B16F10/ErbB2 melanoma cells were left untreated or incubated with 20 ng/mL of recombinant human or murine IFN-γ, respectively, followed by flow cytometric analysis of PD-L1 and ErbB2 expression. (B) Likewise, ectopic overexpression of PD-L1 by lentivirally transduced A549/PD-L1 and Calu-3/PD-L1 cells was confirmed by flow cytometry. Gray lines in (A) and (B) indicate unstained control cells. Figure S7: Effect of IFN-γ treatment on sensitivity of cancer cells toward PD-L1.CAR-expressing NK-92 cells. NCI-N87, KATO III and MDA-MB-453 cells were left untreated or incubated with recombinant human IFN-γ, and then co-cultured with PD-L1.CAR, ErbB2.CAR, PD-L1/ErbB2.CAR or parental NK-92 cells for 2 hours at an E:T ratio or 10:1. Specific lysis was measured using an Europium-based cytotoxicity assay (expanded data sets of the results shown in Figure 4 C). Mean values ± SEM are shown; n=3 independent experiments. ****, p<0.0001; ***, p<0.001; **, p<0.01. Figure S8: A549 and Calu-3 lung cancer cells left untreated (WT), pre-incubated with recombinant human IFN-γ (IFN-γ) or transduced with a PD-L1-encoding lentiviral vector (PD-L1 overexpression) were co-cultured with PD-L1.CAR2, ErbB2.CAR, PD-L1/ErbB2.CAR2 or parental NK-92 cells for 2 hours at the indicated E:T ratios. Specific lysis was determined using a flow cytometry-based cytotoxicity assay. Data were pooled from n=3 independent experiments. Mean values ± SD are shown. ***, p<0.001; **, p<0.01; *, p<0.05. Figure S9: ErbB2.CAR NK-92 cells were pre-incubated for 30 minutes with cytokine-neutralizing antibodies against IFN-γ and/or TNF-α (20 µg/mL) or corresponding isotype controls, as indicated. PD-L1-negative MDA-MB-453 cells were then co-cultured with ErbB2.CAR NK-92 cells for 16 hours at an E:T ratio of 1:10, resulting in a final neutralizing antibody concentration of 10 µg/mL. PD-L1 expression was analyzed by flow cytometry. Data represent mean values ± SEM from three independent experiments. One-way ANOVA was used for statistical analysis. ** p < 0.01; * p < 0.05; ns (not significant) p ≥ 0.05. Figure S10: (A) A549 lung cancer cells were seeded in the bottom chambers of transwell plates and exposed to soluble factors secreted by ErbB2.CAR or PD-L1/ErbB2.CAR2 NK-92 cells cultured in transwell inserts either alone or together with A549 cells to induce effector cell activation. As controls, untreated A549 cells or A549 cells treated with recombinant human IFN-γ were kept in the absence of NK cells. (B) PD-L1 expression on the surface of the cancer cells was measured by flow cytometry as indicated. Representative data from 3 independent experiments are shown. Figure S11: Representative images from the IncuCyte cytotoxicity experiment with MDA-MB-453 breast carcinoma cells co-cultured with dual PD-L1/ErbB2.CAR, PD-L1.CAR, ErbB2.CAR or parental NK-92 cells depicted in Figure 4E. Representative data of time points 0 hours and 72 hours from 3 independent experiments are shown. Figure S12: Representative images of 3D tumor spheroids formed by MDA-MB-453, BxPC-3, and PANC-1 cells expressing red fluorescent protein (Nuclight Red). Cells were cultured as spheroids in Matrigel and co-cultured with the indicated CAR-NK-92 cells at an effector-to-target (E:T) ratio of 1:1. Spheroid growth was monitored over time using an IncuCyte live-cell imaging system. Images shown are representative of three independent experiments. Figure S13: (A) PD-L1.CAR, ErbB2.CAR, PD-L1/ErbB2.CAR, and parental NK-92 cells were analyzed for PD-L1 expression by flow cytometry. An irrelevant isotype-matched antibody was used as a negative control, and MDA-MB-231 cells served as a positive control. (B) Quantification of PD-L1 expression as mean fluorescence intensity (MFI), as shown in (A), pooled from three independent experiments. (C) Viability of PD-L1.CAR, ErbB2.CAR, PD-L1/ErbB2.CAR, and parental NK-92 cells was assessed by trypan blue exclusion. Data represent mean values ± SEM (n = 10). One-way ANOVA was used for statistical analysis. ****, p<0.0001; ns (not significant) p≥0.05. Figure S14: (A) PBMCs and MSCs from healthy donors were analyzed for PD-L1 and ErbB2 expression by flow cytometry. Gray lines indicate isotype controls. Representative data from three donors are shown. (B) PBMCs and MSCs were co-cultured with PD-L1/ErbB2.CAR NK-92 cells and corresponding controls, as indicated, for 2 hours, and cytotoxicity was measured using a Europium-based cytotoxicity assay. Pooled data from three donors are shown as mean values ± SEM. Figure S15: (A) Flow cytometry analysis of PD-L1.CAR and ErbB2.CAR expression on primary blood-derived NK cells transduced with PD-L1.CAR alone, ErbB2.CAR alone, or co-transduced with both PD-L1.CAR and ErbB2.CAR (dual PD-L1/ErbB2.CAR). Data are pre-gated on viable CD3⁻ CD56⁺ cells. (B) Cytotoxicity of untransduced, single-transduced (PD-L1.CAR or ErbB2.CAR), or co-transduced dual PD-L1/ErbB2.CAR primary NK cells against MDA-MB-231 cells at an effector-to-target (E:T) ratio of 1:10. Representative data from two healthy donors are shown. Two-way ANOVA was used for statistical analysis. ****, p<0.0001; *, p<0.05; ns (not significant) p≥0.05. Figure S16: Activity of CAR-NK cells against ascites cells from ovarian cancer patients. (A) Gating strategy for the identification of ErbB2⁺ or PD-L1⁺ cells. (B) Frequency of ErbB2⁺ or PD-L1⁺ cells in ascites cultured alone or co-cultured for 6 hours at an E:T ratio of 1:1 with NK-92 cells or CAR-NK cells, as indicated. Mean values ± SEM are shown; n=5. Figure S17: Flow cytometry cytotoxicity plots of ascites-derived cancer cells co-cultured with CAR-NK-92 cells at the indicated effector-to-target (E:T) ratios. Upper panels show results at an E:T ratio of 1:1, and lower panels at an E:T ratio of 3:1. Figure S18: (A) Primary cancer cells were isolated from an ovarian cancer patient, immunomagnetically enriched for tumor cells, and then analyzed for ErbB2 and PD-L1 expression by flow cytometry as indicated. Gray lines indicate isotype control. (B) Sensitivity of the primary ovarian cancer cells to CAR-NK cells was analyzed by co-incubation with dual PD-L1/ErbB2.CAR, ErbB2.CAR or parental NK-92 cells for 2 hours at an E:T ratio of 10:1, and specific cytotoxicity was measured using an Europium-based assay. Mean values ± SD are shown. One-way ANOVA was used for statistical analysis. ***, p<0.001; **, p<0.01. Figure S19: In vivo activity of CAR-NK cells against breast carcinoma xenografts. Shown are images with bioluminescence signals taken at the indicated time points of mice injected with MDA-MB-231/Luc/ErbB2 and the indicated NK-92 derivatives. Quantitative data are depicted in Figure 7F

## Data Availability

All data relevant to the study are included in the article or uploaded as online Supplementary Material. Further inquiries can be directed to the corresponding authors.
