# Supplementary material for: Dual CAR-NK cells targeting PD-L1 and ErbB2 (HER2) exhibit cooperative CAR signaling and counteract solid tumor heterogeneity
Source: J Exp Clin Cancer Res. 2026 May 19;45:127. doi: 10.1186/s13046-026-03722-6 (PMC13198055; doi:10.1186/s13046-026-03722-6)
Supplement: Supplementary file 1 — Supplementary Material 1: Figure S1: (A) CAR expression on PD-L1/ErbB2.CAR, PD-L1.CAR and ErbB2.CAR NK-92 cells was determined by flow cytometry using recombinant ErbB2-Fc or PD-L1-Fc protein, followed by staining with an anti-Fc secondary antibody. Parental NK-92 cells were included for comparison. (B) CAR expression on PD-L1/ErbB2.CAR2, PD-L1.CAR2 and ErbB2.CAR NK-92 cells was determined by flow cytometry. Filled gray areas indicate negative controls only stained with secondary antibody. Representative data from at least 3 independent experiments are shown. Figure S2: (A) Gating strategy applied for the flow cytometric protein phosphorylation analysis shown in Figures 3 and S3. Shown are representative data upon co-culture of NK cells with MDA-MB-231 cancer cells. Singlets were selected based on FSC and SSC parameters. NK cells were identified by CD56 staining, and further analyzed with anti-phosphoprotein antibodies as depicted in Figures 3 A and S3. (B,C) Flow cytometry data histograms showing expression of PD-L1 (blue) and ErbB2 (red) by BXPC-3 ErbB2 OE (ErbB2 overexpressed), Calu-3 PDL1 OE (PD-L1 overexpressed), MDA-MB-468 (WT), MDA-MB-468 PD-L1 OE (PD-L1 overexpressed), MDA-MB-468 ErbB2 OE (ErbB2 overexpressed), and MDA-MB-468 PD-L1/ErbB2 OE (PD-L1 and ErbB2 overexpressed) cells. Figure S3: Downstream PLCγ and PI3K signaling in dual PD-L1/ErbB2.CAR NK-92 cells. PD-L1/ErbB2.CAR, PD-L1.CAR, ErbB2.CAR or parental NK-92 cells were co-cultured with PD-1/ErbB2 double-positive MDA-MB-231 breast carcinoma cells for 20 or 60 min, or kept without target cells. Phosphorylation of the indicated signaling molecules in NK cells was determined by flow cytometry. Activation of PLCγ and PI3K pathways was analyzed by assessing pPLCγ1 (Ser1248) and pAkt (Ser473), respectively. (A) Histograms from a representative experiment depicting NK cells in the presence (red) or absence (blue) of target cells. (B) Mean fluorescence intensity (MFI) data from n=3 independent experiments. Mea [file 13046_2026_3722_MOESM1_ESM.pdf]

Fig. S1

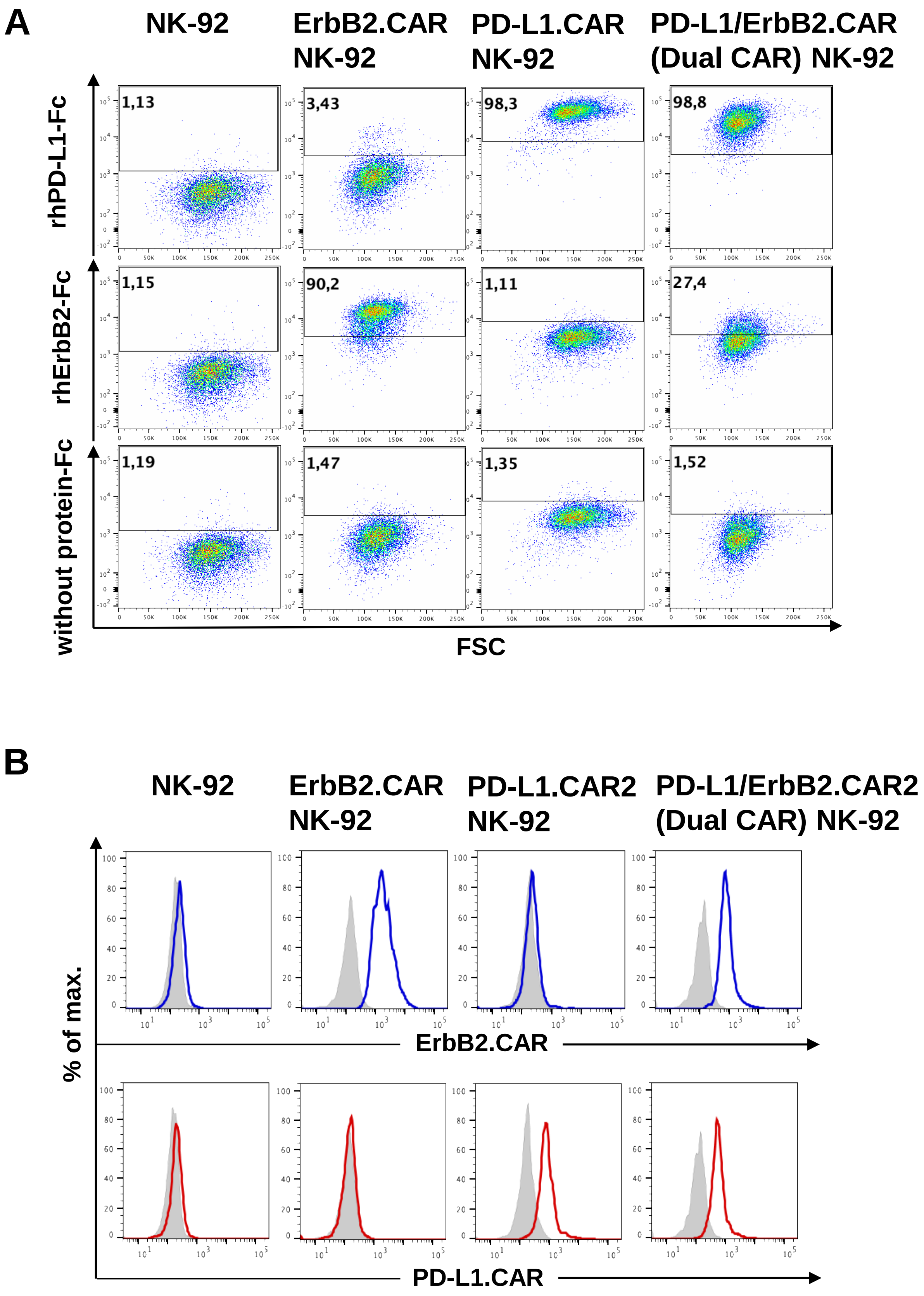
A
NK-92
ErbB2.CAR NK-92
PD-L1.CAR NK-92
PD-L1/ErbB2.CAR (Dual CAR) NK-92
rhPD-L1-Fc
rhErbB2-Fc
without protein-Fc
1,13
3,43
98,3
98,8
1,15
90,2
1,11
27,4
1,19
1,47
1,35
1,52
FSC
B
NK-92
ErbB2.CAR NK-92
PD-L1.CAR2 NK-92
PD-L1/ErbB2.CAR2 (Dual CAR) NK-92
% of max.
ErbB2.CAR
PD-L1.CAR

Fig. S2

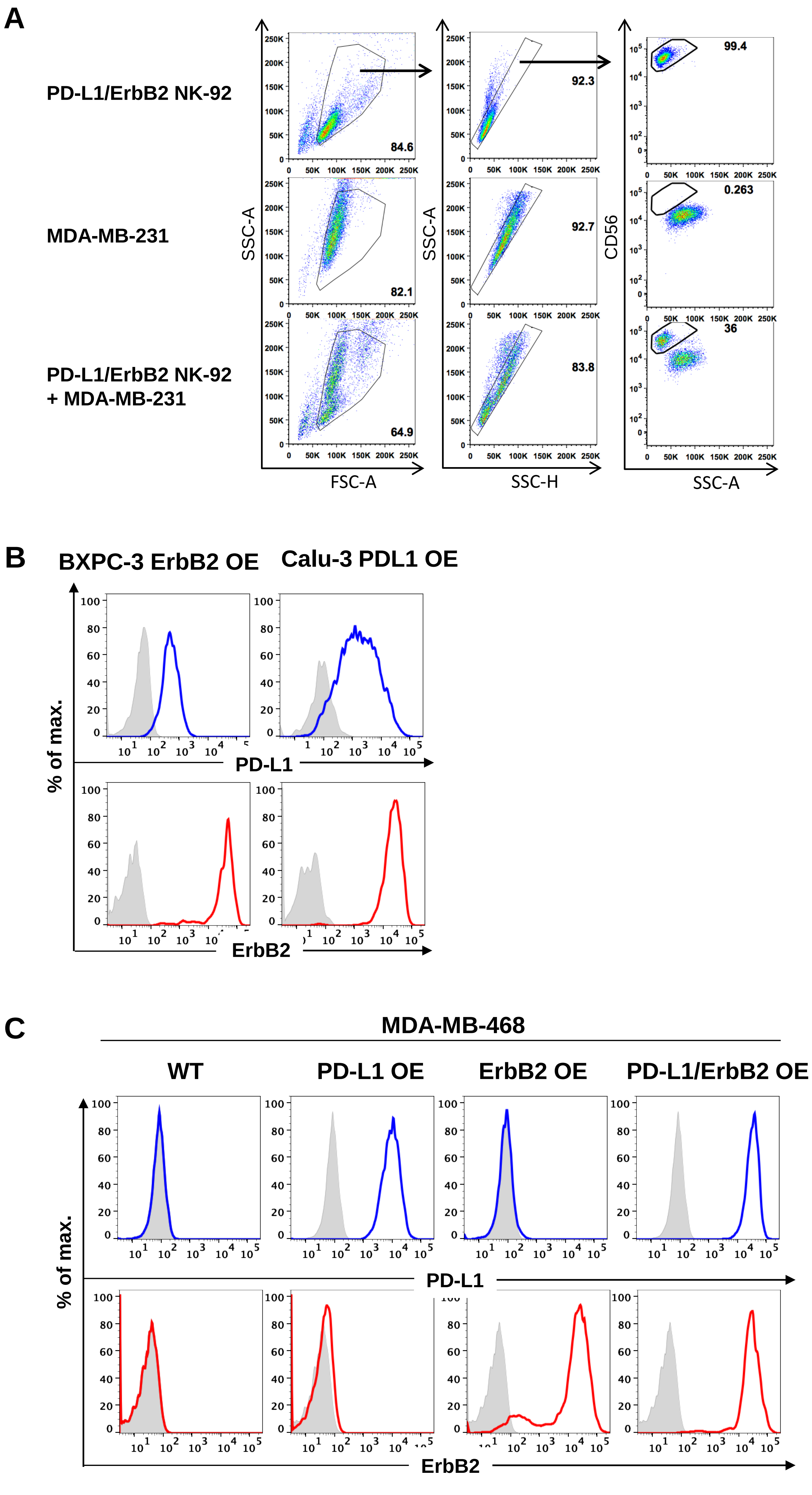

A
PD-L1/ErbB2 NK-92
MDA-MB-231
PD-L1/ErbB2 NK-92
+ MDA-MB-231
SSC-A
FSC-A
SSC-H
CD56
84.6
82.1
64.9
92.3
92.7
83.8
99.4
0.263
36
B
BXPC-3 ErbB2 OE
Calu-3 PDL1 OE
% of max.
PD-L1
ErbB2
C
MDA-MB-468
WT
PD-L1 OE
ErbB2 OE
PD-L1/ErbB2 OE

Fig. S3

A

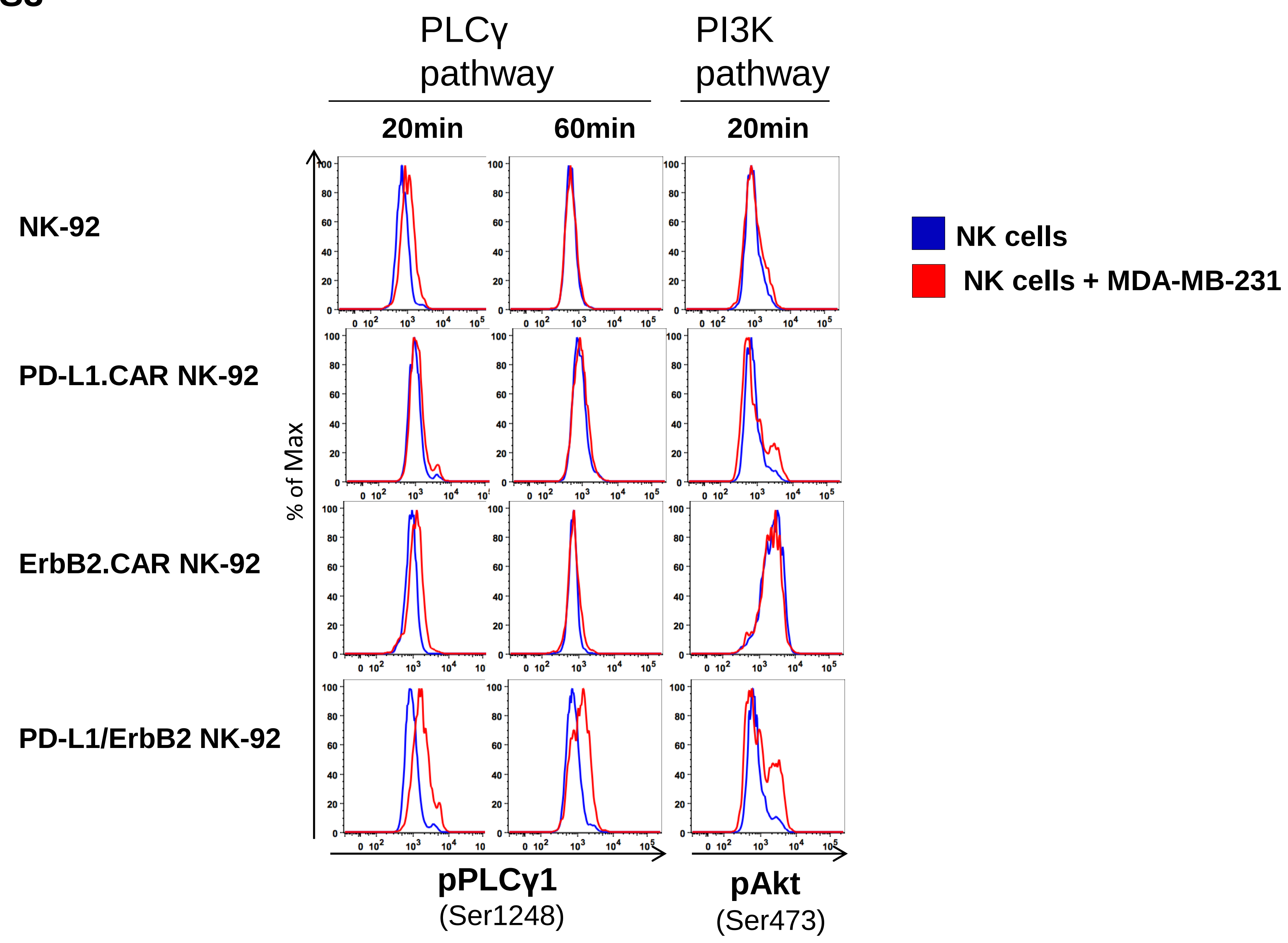
PLCγ pathway
PI3K pathway
20min
60min
20min
NK-92
PD-L1.CAR NK-92
ErbB2.CAR NK-92
PD-L1/ErbB2 NK-92
% of Max
NK cells
NK cells + MDA-MB-231
pPLCγ1
(Ser1248)
pAkt
(Ser473)

B

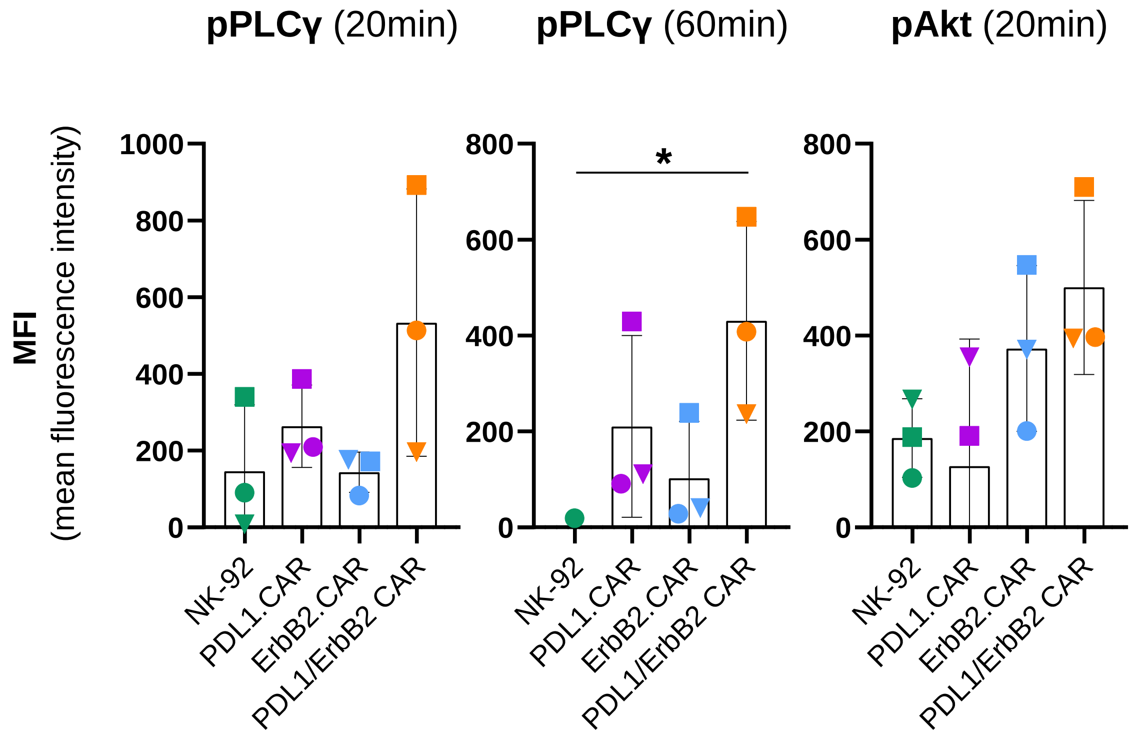
pPLCγ (20min)
pPLCγ (60min)
pAkt (20min)
MFI
(mean fluorescence intensity)
*
NK-92
PDL1.CAR
ErbB2.CAR
PDL1/ErbB2 CAR

Fig. S4

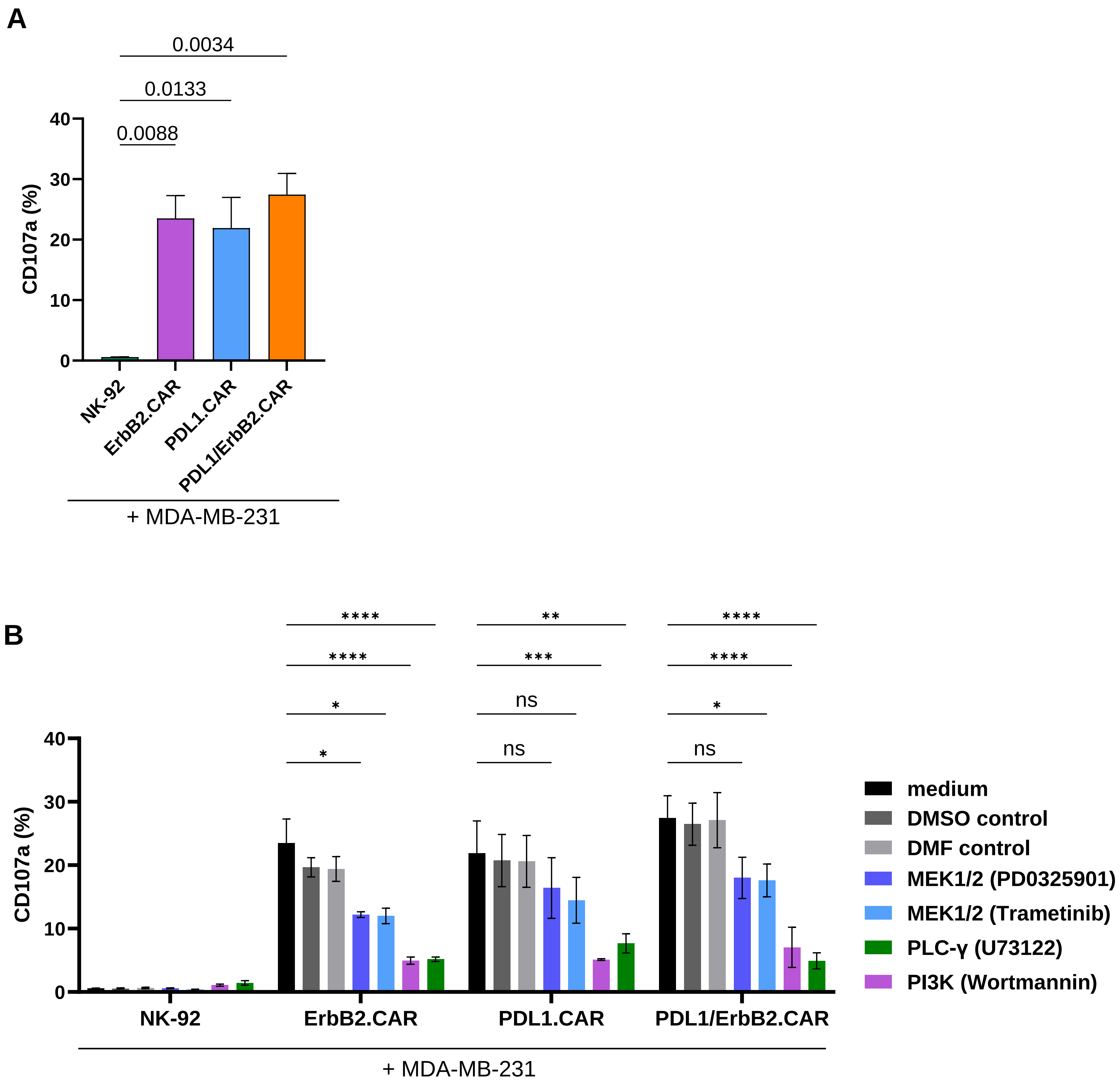
A
0.0034
0.0133
0.0088
CD107a (%)
40
30
20
10
0
NK-92
ErbB2.CAR
PDL1.CAR
PDL1/ErbB2.CAR
+ MDA-MB-231
B
****
**
****
****
***
****
*
ns
*
*
ns
ns
CD107a (%)
40
30
20
10
0
NK-92
ErbB2.CAR
PDL1.CAR
PDL1/ErbB2.CAR
+ MDA-MB-231
medium
DMSO control
DMF control
MEK1/2 (PD0325901)
MEK1/2 (Trametinib)
PLC-γ (U73122)
PI3K (Wortmannin)

Fig. S5

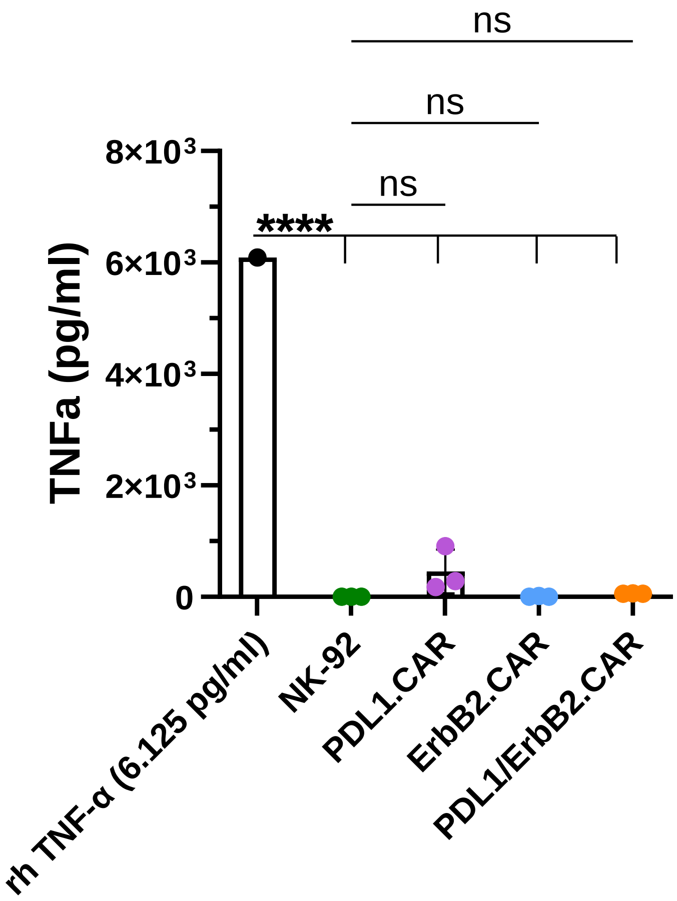
ns
ns
ns
****
8×10³
6×10³
4×10³
2×10³
0
TNFa (pg/ml)
rh TNF-α (6.125 pg/ml)
NK-92
PDL1.CAR
ErbB2.CAR
PDL1/ErbB2.CAR

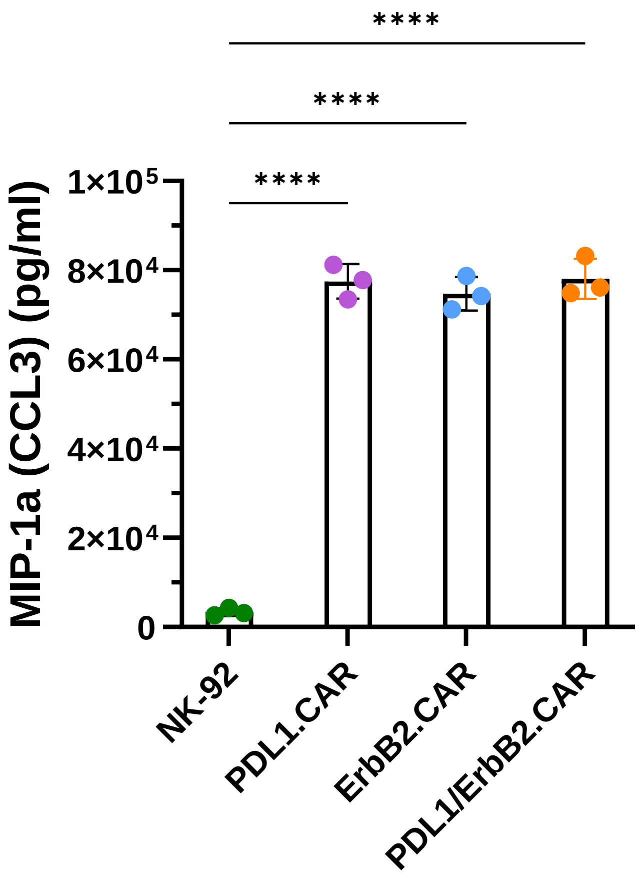
****
****
****
1×10⁵
8×10⁴
6×10⁴
4×10⁴
2×10⁴
0
MIP-1a (CCL3) (pg/ml)
NK-92
PDL1.CAR
ErbB2.CAR
PDL1/ErbB2.CAR

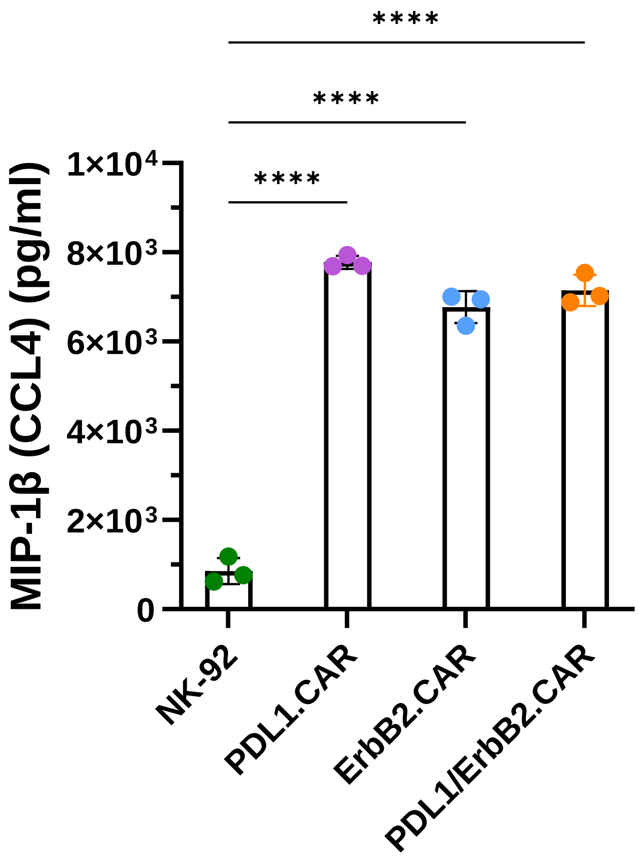
****
****
****
1×10⁴
8×10³
6×10³
4×10³
2×10³
0
MIP-1β (CCL4) (pg/ml)
NK-92
PDL1.CAR
ErbB2.CAR
PDL1/ErbB2.CAR

Fig. S6

A

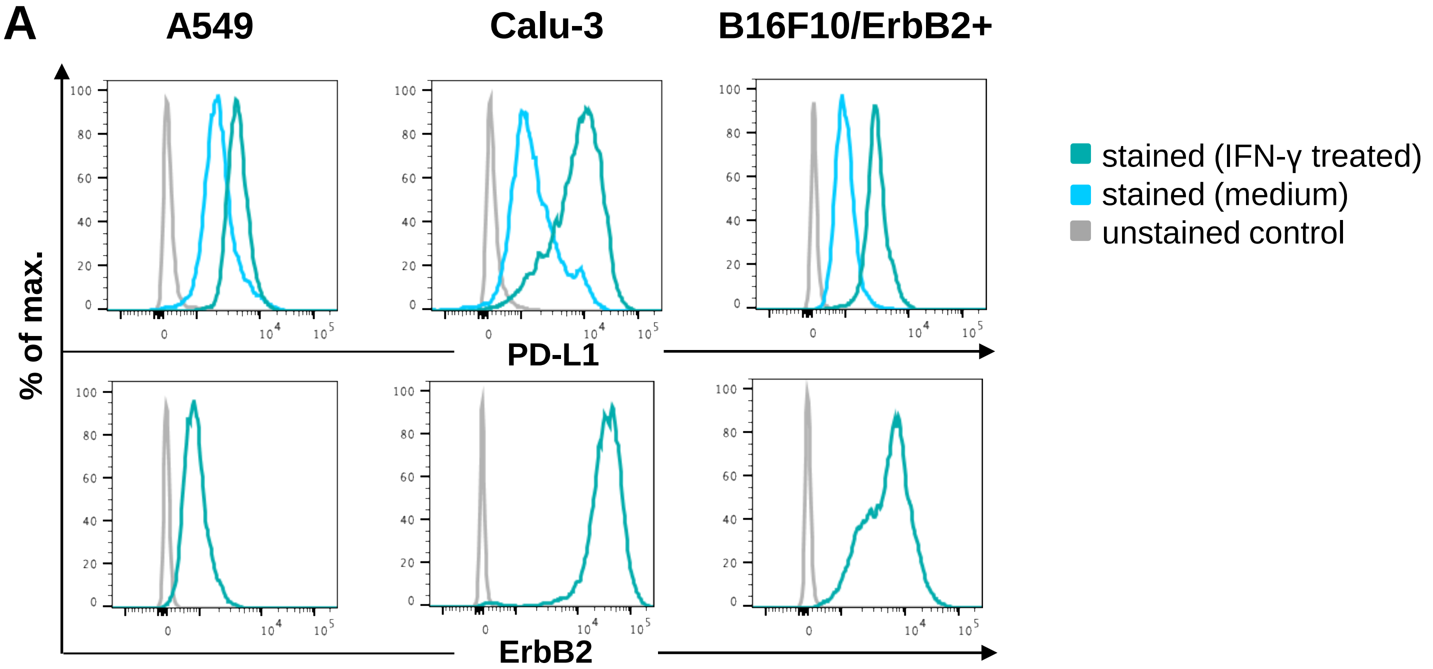
A549
Calu-3
B16F10/ErbB2+
% of max.
PD-L1
ErbB2
stained (IFN-γ treated)
stained (medium)
unstained control

B

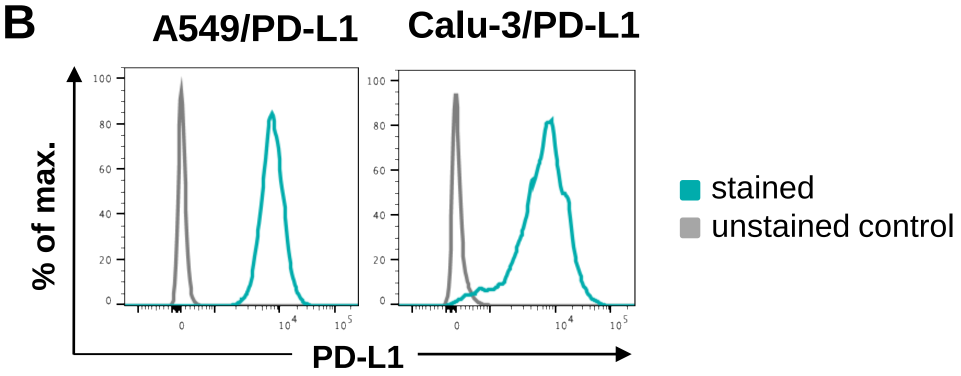
A549/PD-L1
Calu-3/PD-L1
% of max.
PD-L1
stained
unstained control

Fig. S7

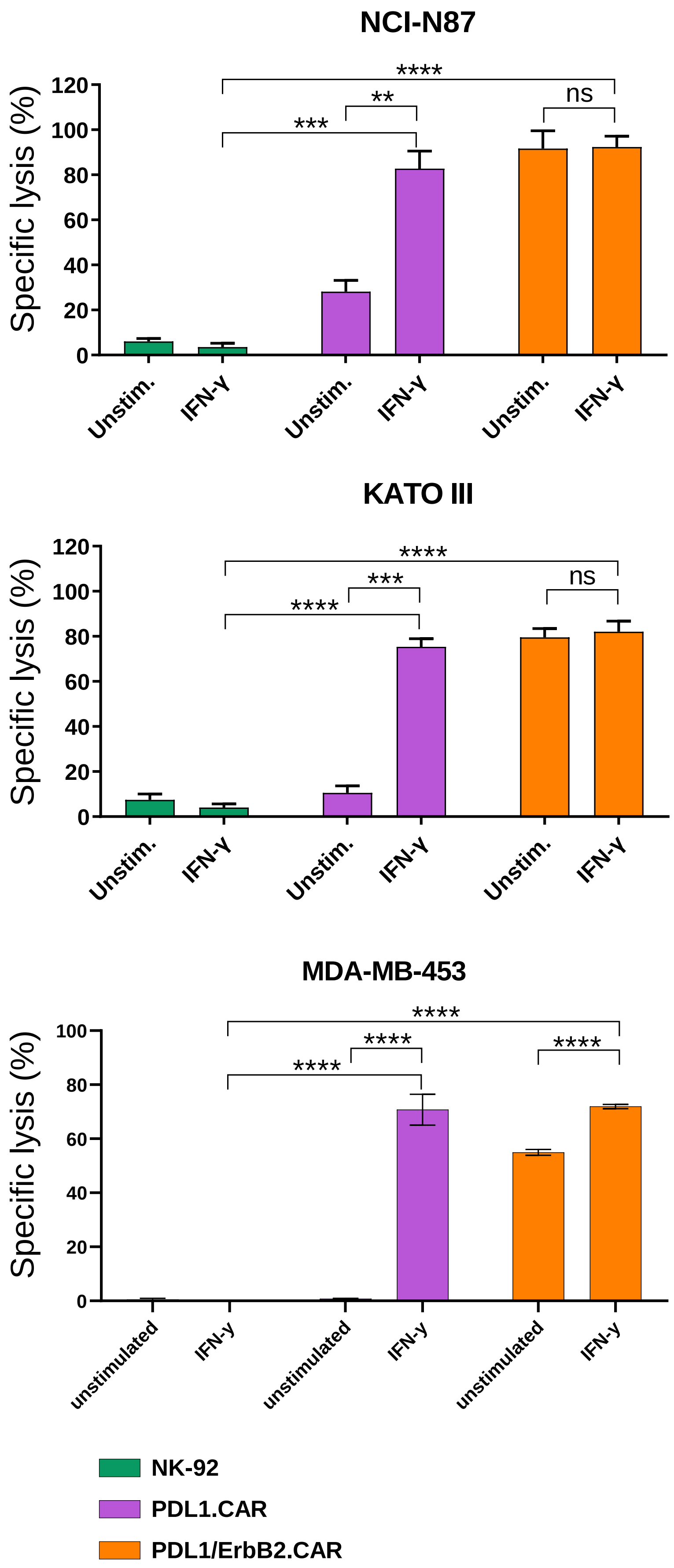
NCI-N87
Specific lysis (%)
0
20
40
60
80
100
120
****
**
***
ns
Unstim.
IFN-γ
Unstim.
IFN-γ
Unstim.
IFN-γ
KATO III
Specific lysis (%)
0
20
40
60
80
100
120
****
***
****
ns
Unstim.
IFN-γ
Unstim.
IFN-γ
Unstim.
IFN-γ
MDA-MB-453
Specific lysis (%)
0
20
40
60
80
100
****
****
****
****
unstimulated
IFN-γ
unstimulated
IFN-γ
unstimulated
IFN-γ
NK-92
PDL1.CAR
PDL1/ErbB2.CAR

Fig. S8

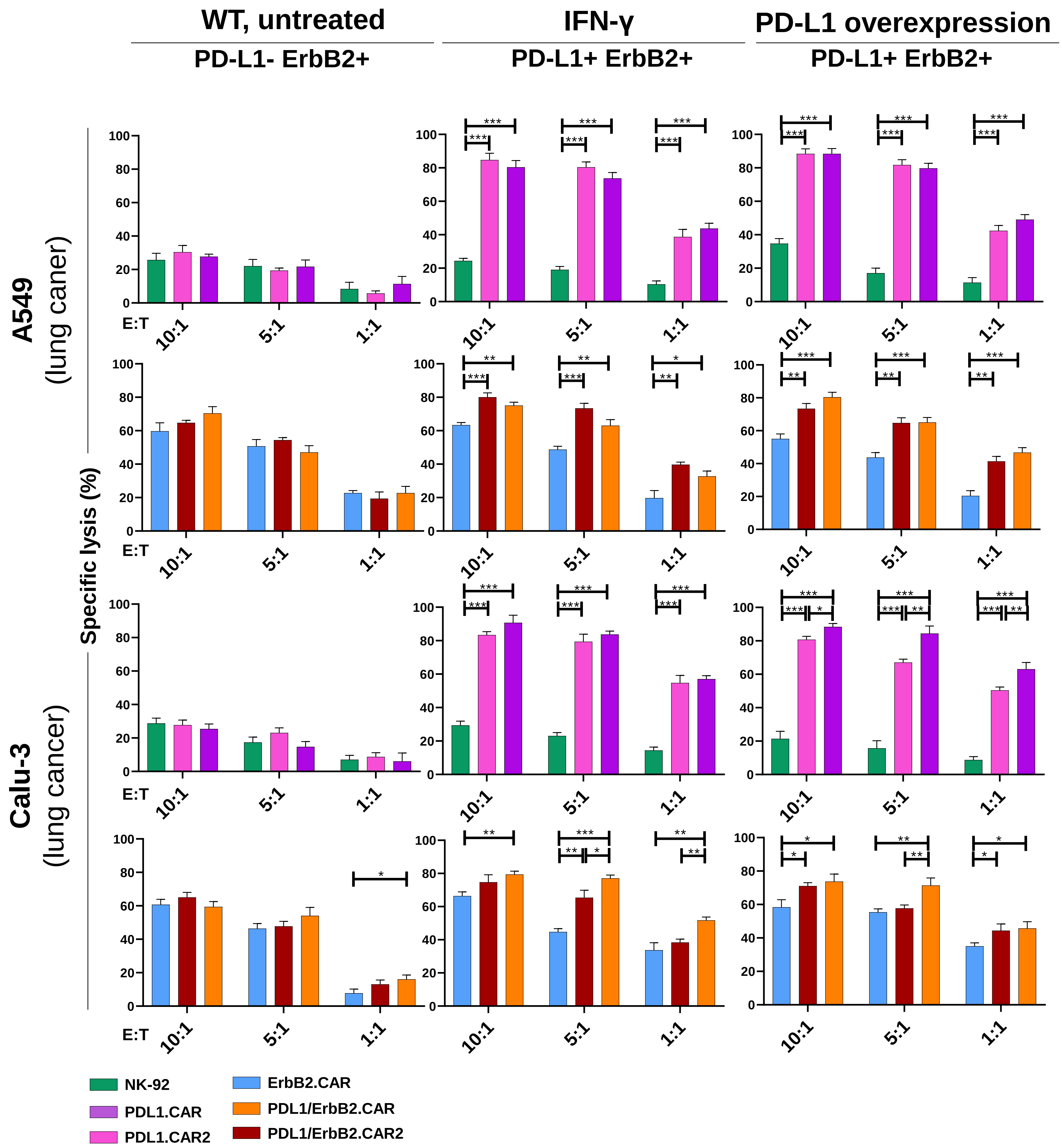

WT, untreated
PD-L1- ErbB2+
IFN-γ
PD-L1+ ErbB2+
PD-L1 overexpression
PD-L1+ ErbB2+
A549
(lung caner)
Calu-3
(lung cancer)
Specific lysis (%)
E:T
10:1
5:1
1:1
NK-92
PDL1.CAR
PDL1.CAR2
ErbB2.CAR
PDL1/ErbB2.CAR
PDL1/ErbB2.CAR2

Fig. S9

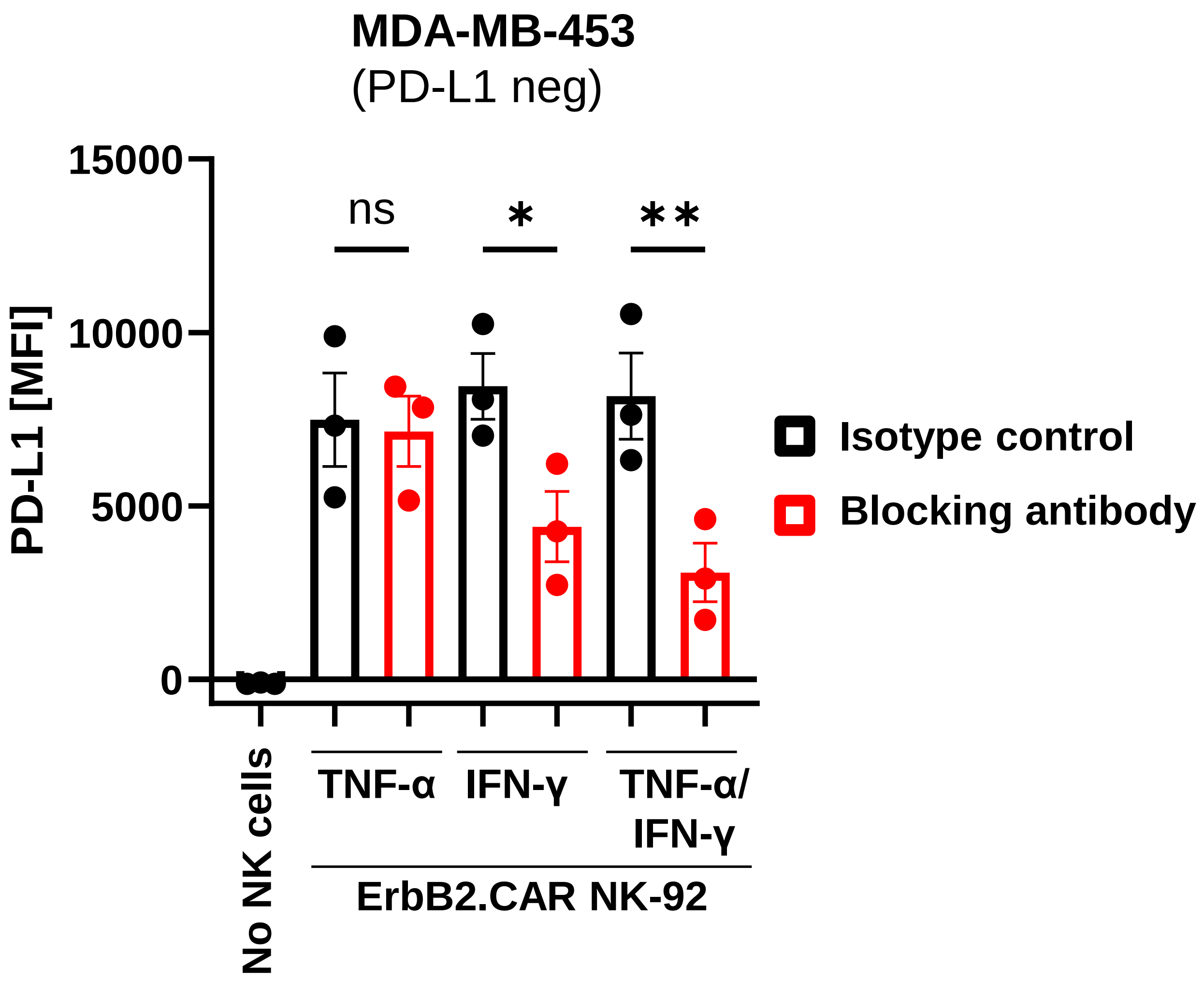

MDA-MB-453
(PD-L1 neg)
PD-L1 [MFI]
15000
10000
5000
0
ns
*
**
Isotype control
Blocking antibody
No NK cells
TNF-α
IFN-γ
TNF-α/
IFN-γ
ErbB2.CAR NK-92

Fig. S10

A

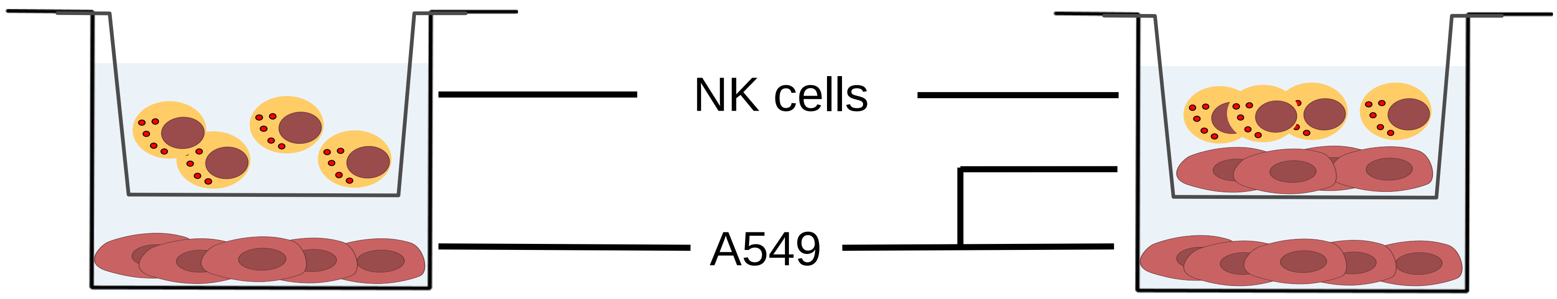
NK cells
A549

B

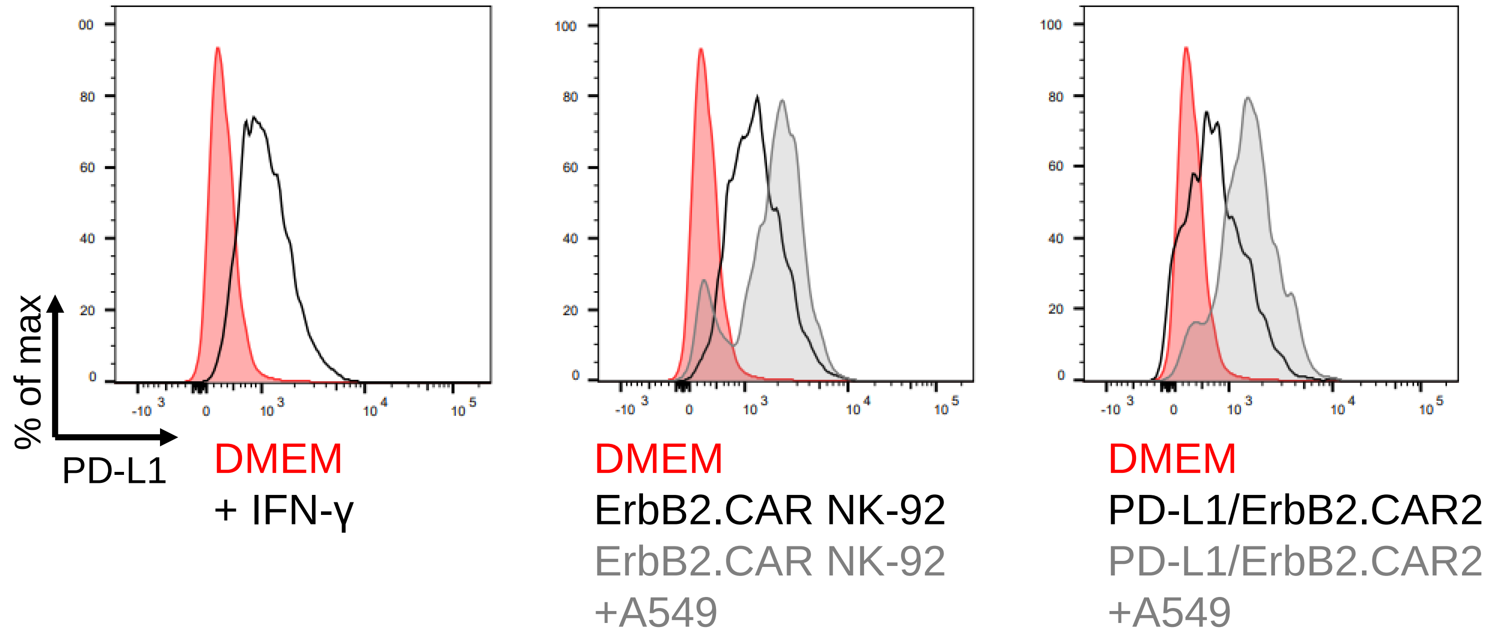
% of max
PD-L1
DMEM
+ IFN-γ
DMEM
ErbB2.CAR NK-92
ErbB2.CAR NK-92
+A549
DMEM
PD-L1/ErbB2.CAR2
PD-L1/ErbB2.CAR2
+A549

Fig. S11

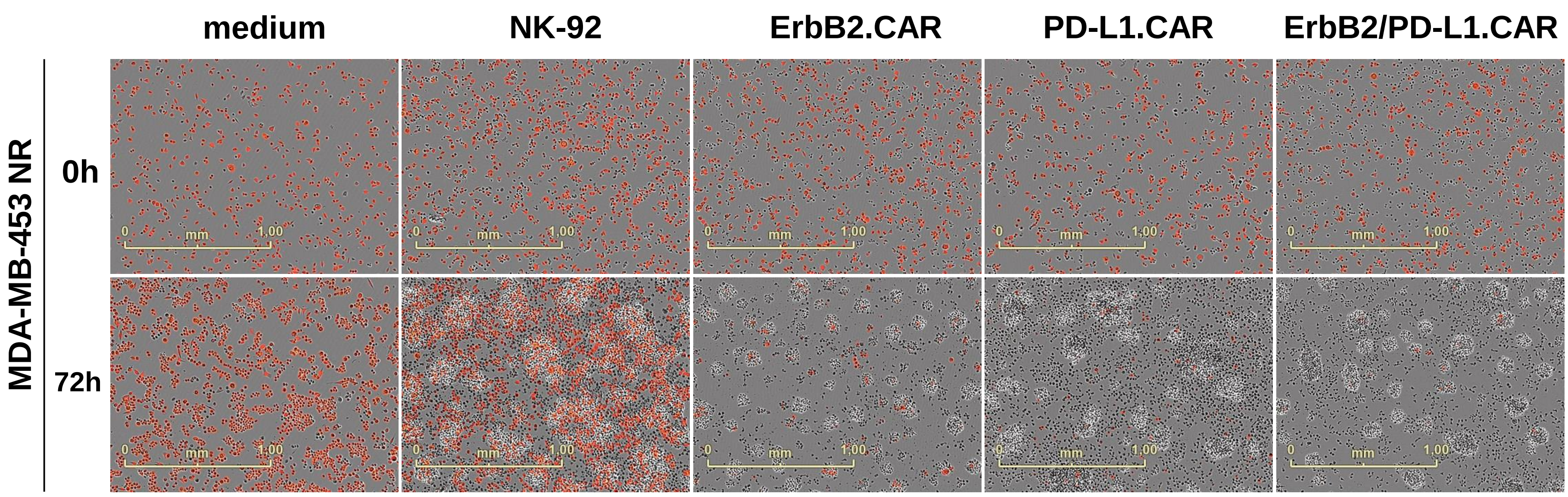
medium
NK-92
ErbB2.CAR
PD-L1.CAR
ErbB2/PD-L1.CAR
MDA-MB-453 NR
0h
72h

Fig. S12

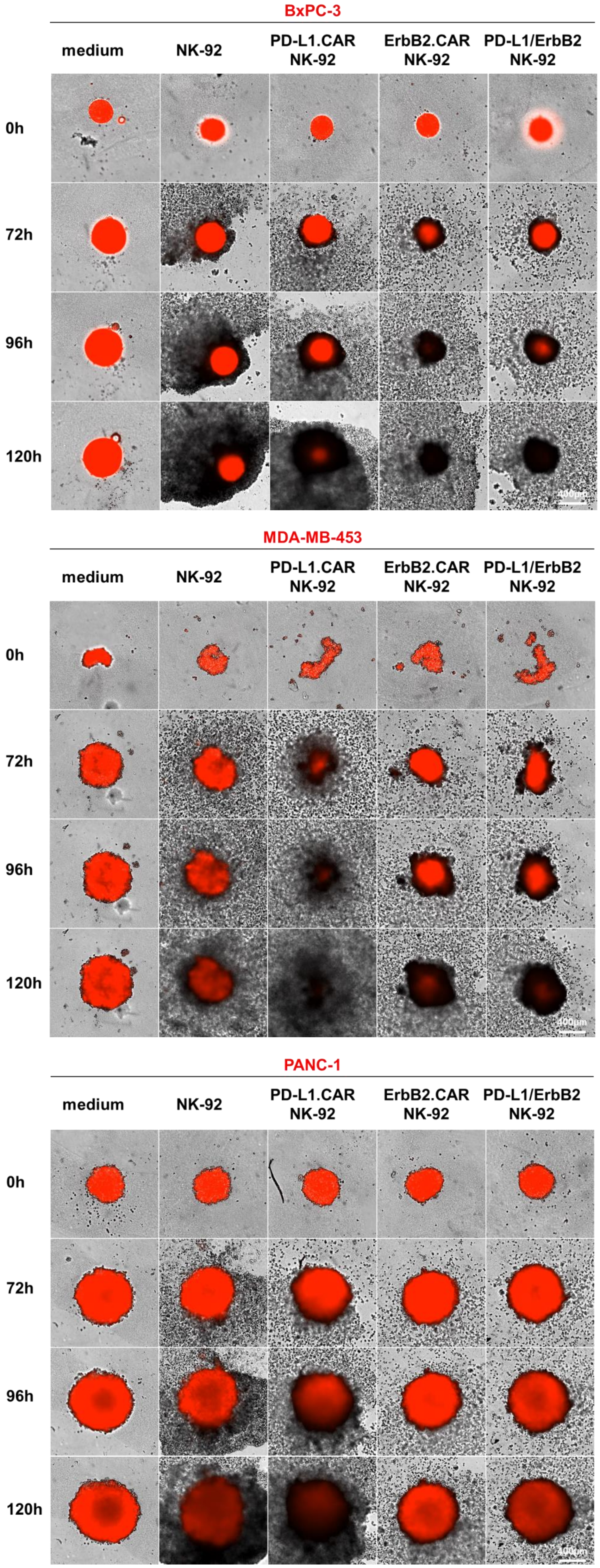
BxPC-3
medium
NK-92
PD-L1.CAR NK-92
ErbB2.CAR NK-92
PD-L1/ErbB2 NK-92
0h
72h
96h
120h
MDA-MB-453
medium
NK-92
PD-L1.CAR NK-92
ErbB2.CAR NK-92
PD-L1/ErbB2 NK-92
0h
72h
96h
120h
PANC-1
medium
NK-92
PD-L1.CAR NK-92
ErbB2.CAR NK-92
PD-L1/ErbB2 NK-92
0h
72h
96h
120h

Fig. S13

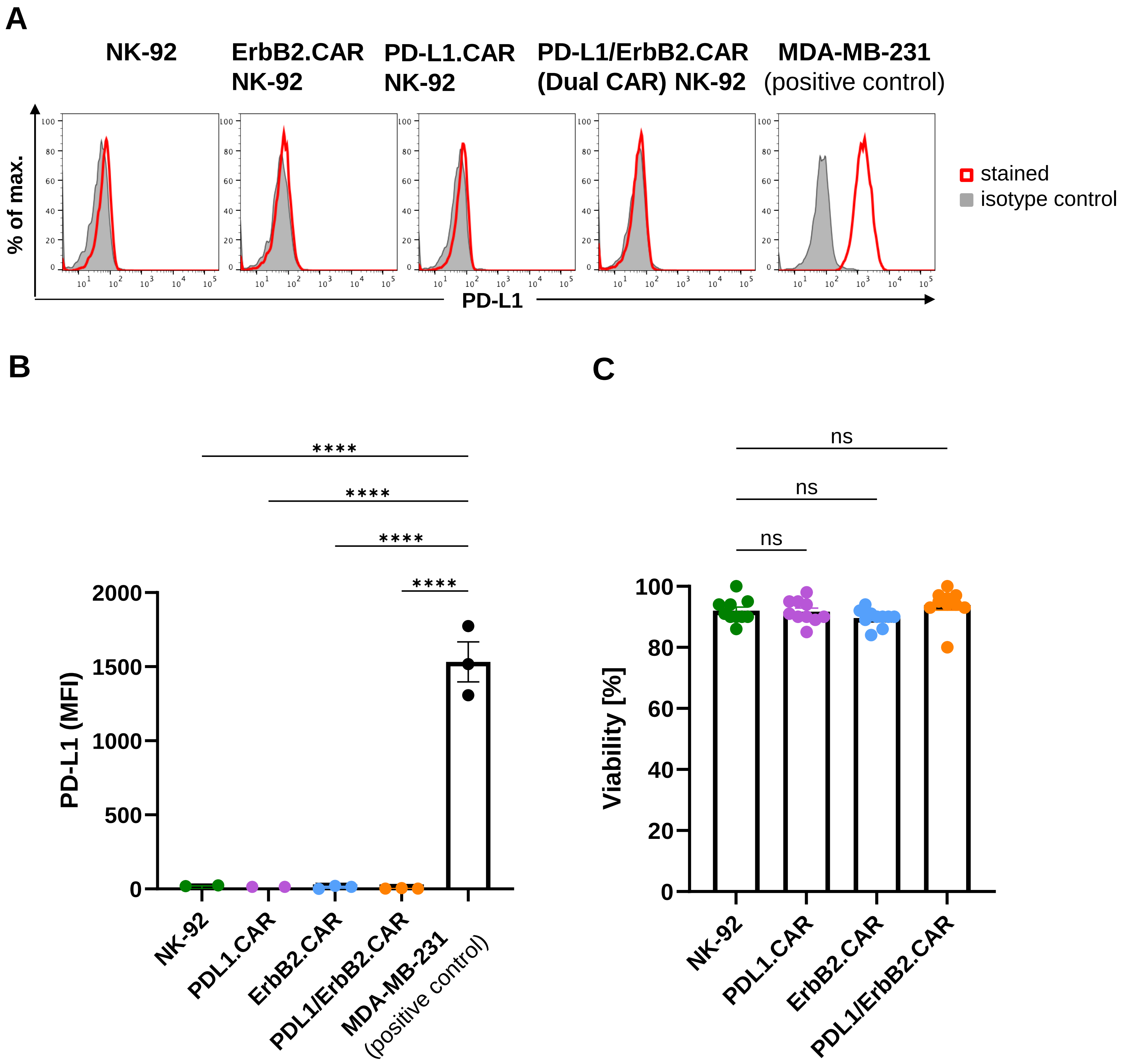
A
NK-92
ErbB2.CAR NK-92
PD-L1.CAR NK-92
PD-L1/ErbB2.CAR (Dual CAR) NK-92
MDA-MB-231 (positive control)
% of max.
PD-L1
stained
isotype control
B
PD-L1 (MFI)
2000
1500
1000
500
0
****
****
****
****
NK-92
PDL1.CAR
ErbB2.CAR
PDL1/ErbB2.CAR
MDA-MB-231 (positive control)
C
Viability [%]
100
80
60
40
20
0
ns
ns
ns
NK-92
PDL1.CAR
ErbB2.CAR
PDL1/ErbB2.CAR

Fig. S14

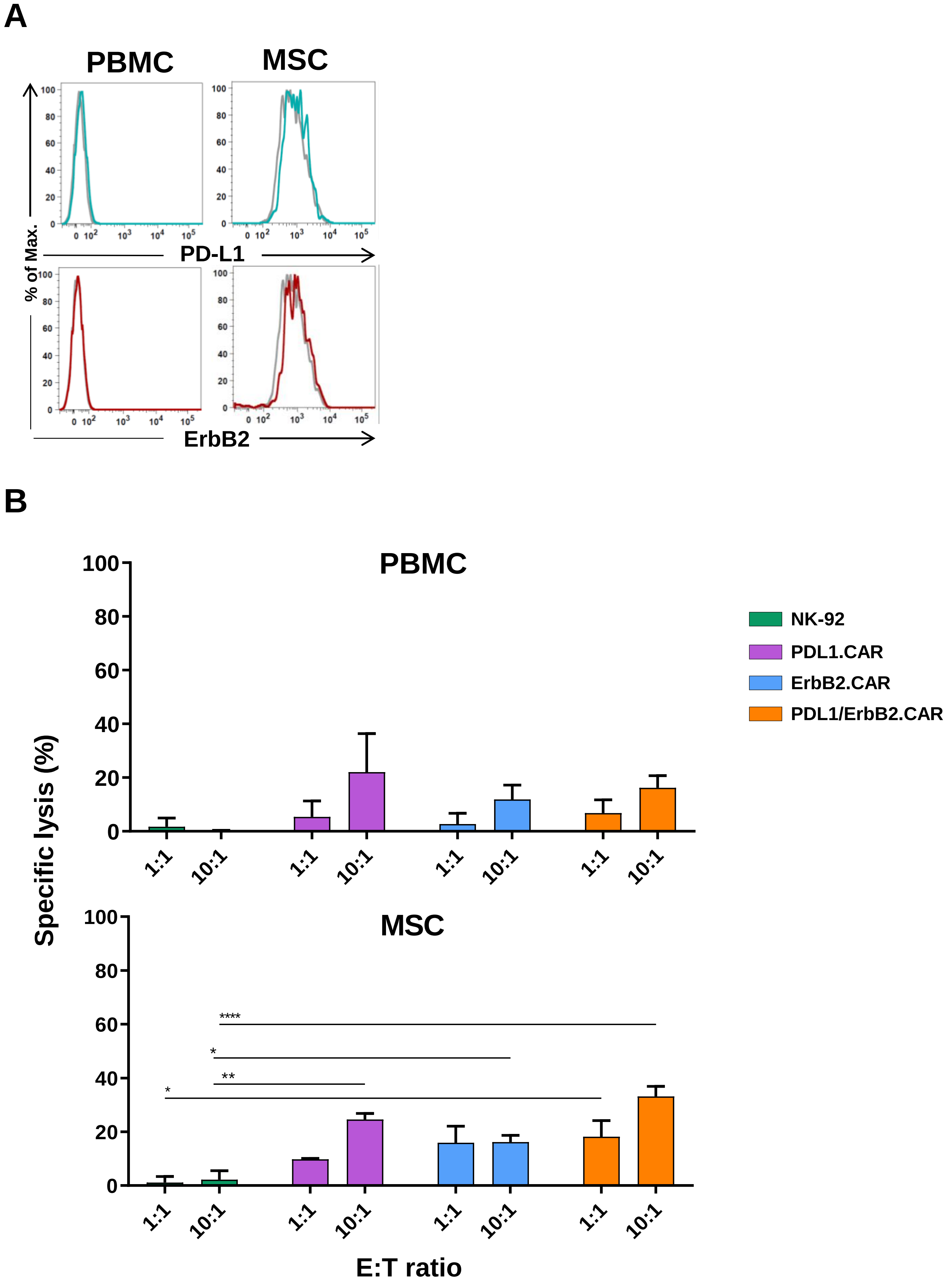
A
PBMC
MSC
% of Max.
PD-L1
ErbB2
B
PBMC
MSC
Specific lysis (%)
E:T ratio
1:1
10:1
NK-92
PDL1.CAR
ErbB2.CAR
PDL1/ErbB2.CAR
****
*
**
*

Fig. S15

A

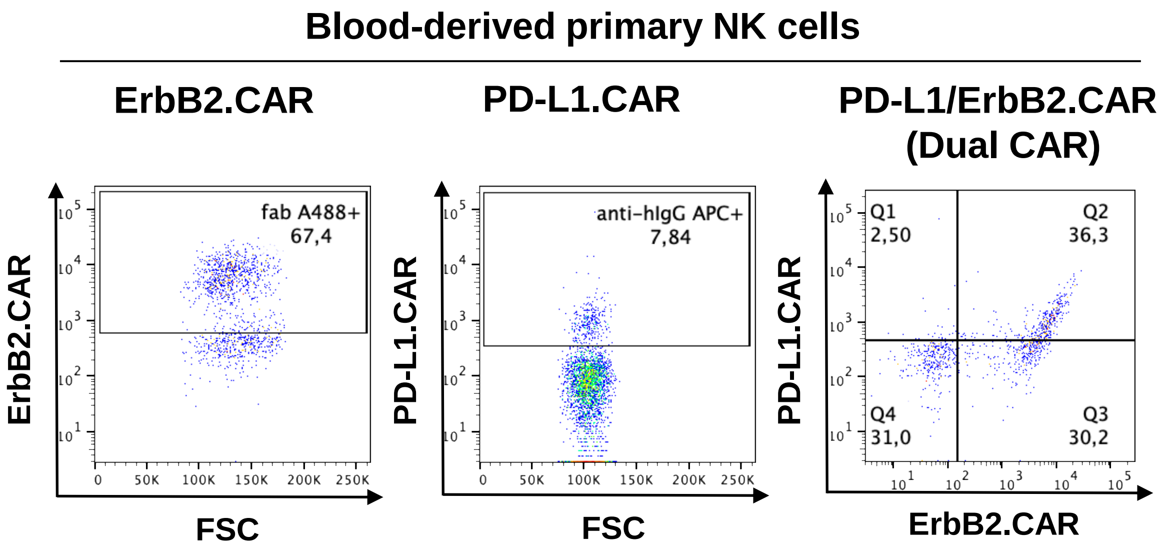

Blood-derived primary NK cells
ErbB2.CAR
PD-L1.CAR
PD-L1/ErbB2.CAR
(Dual CAR)
fab A488+
67,4
anti-hIgG APC+
7,84
Q1
2,50
Q2
36,3
Q4
31,0
Q3
30,2
ErbB2.CAR
PD-L1.CAR
FSC

B

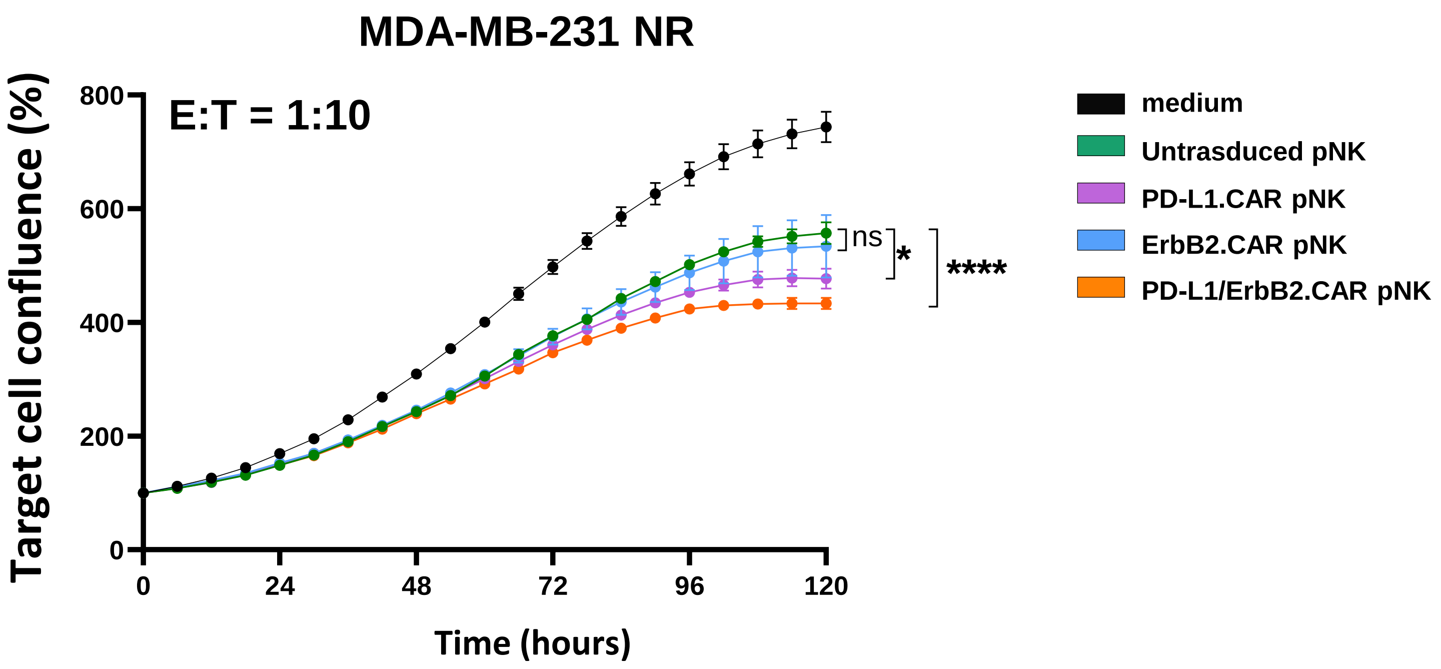

MDA-MB-231 NR
E:T = 1:10
Target cell confluence (%)
Time (hours)
ns
*
****
medium
Untrasduced pNK
PD-L1.CAR pNK
ErbB2.CAR pNK
PD-L1/ErbB2.CAR pNK

Fig. S16

A

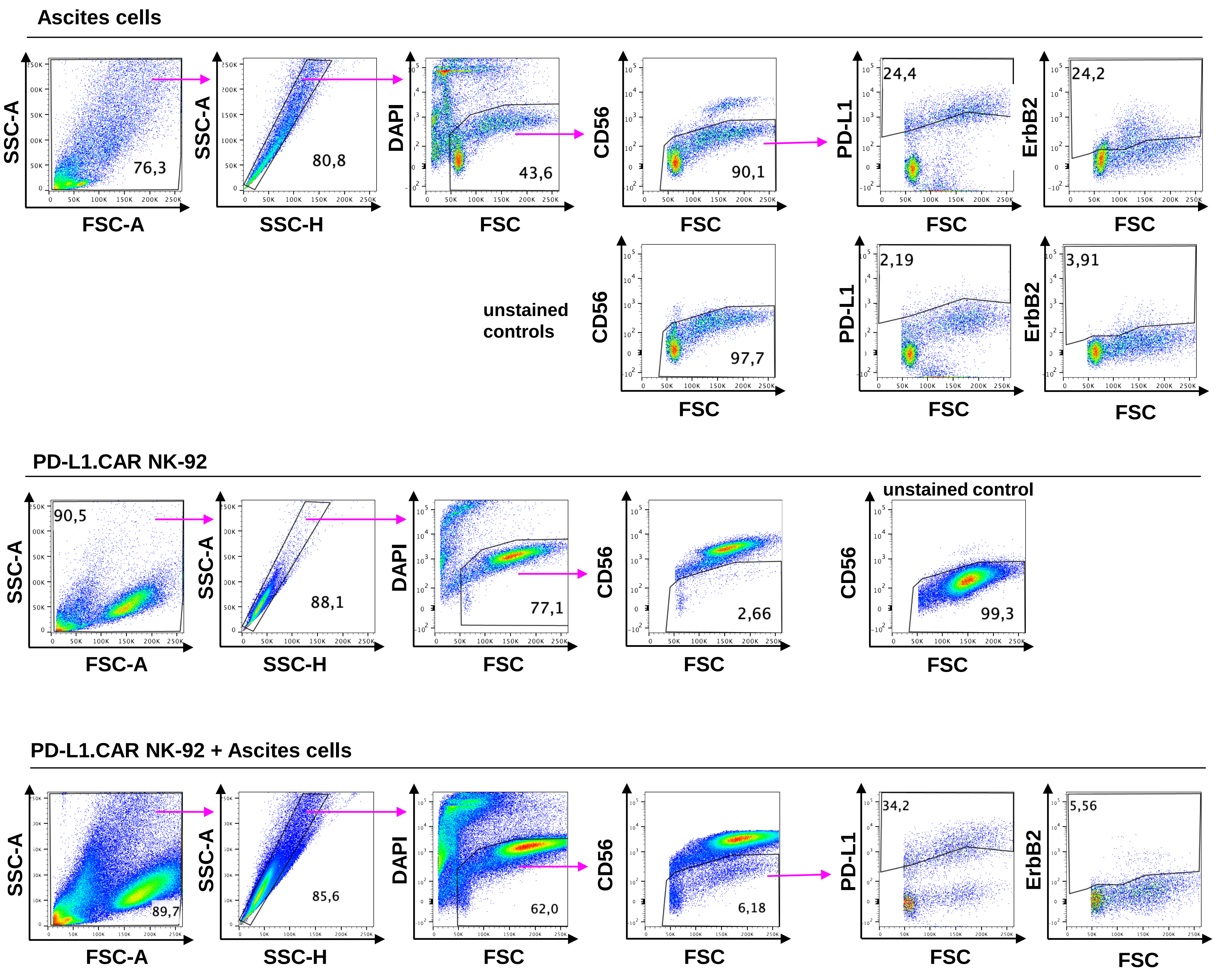

Ascites cells
SSC-A
FSC-A
76,3
SSC-H
80,8
DAPI
FSC
43,6
CD56
90,1
PD-L1
24,4
ErbB2
24,2
unstained controls
97,7
2,19
3,91
PD-L1.CAR NK-92
90,5
88,1
77,1
2,66
unstained control
99,3
PD-L1.CAR NK-92 + Ascites cells
89,7
85,6
62,0
6,18
34,2
5,56

B

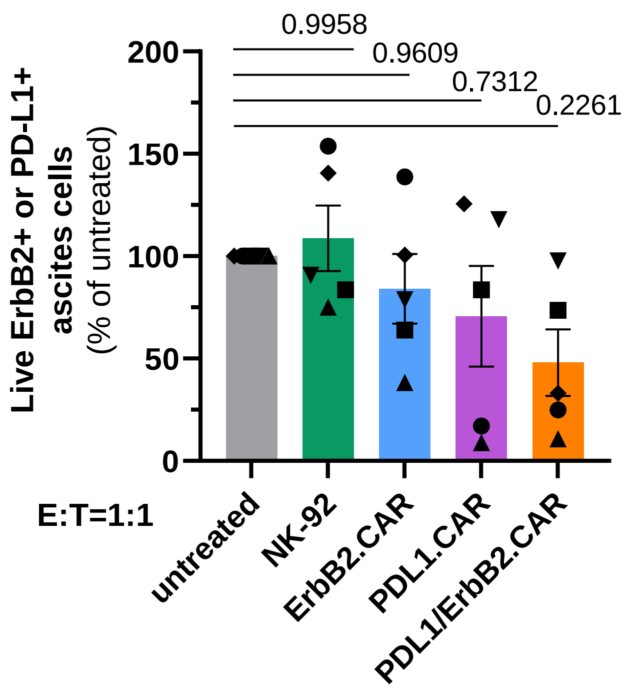

Live ErbB2+ or PD-L1+ ascites cells
(% of untreated)
200
150
100
50
0
0.9958
0.9609
0.7312
0.2261
E:T=1:1
untreated
NK-92
ErbB2.CAR
PDL1.CAR
PDL1/ErbB2.CAR

Fig. S17

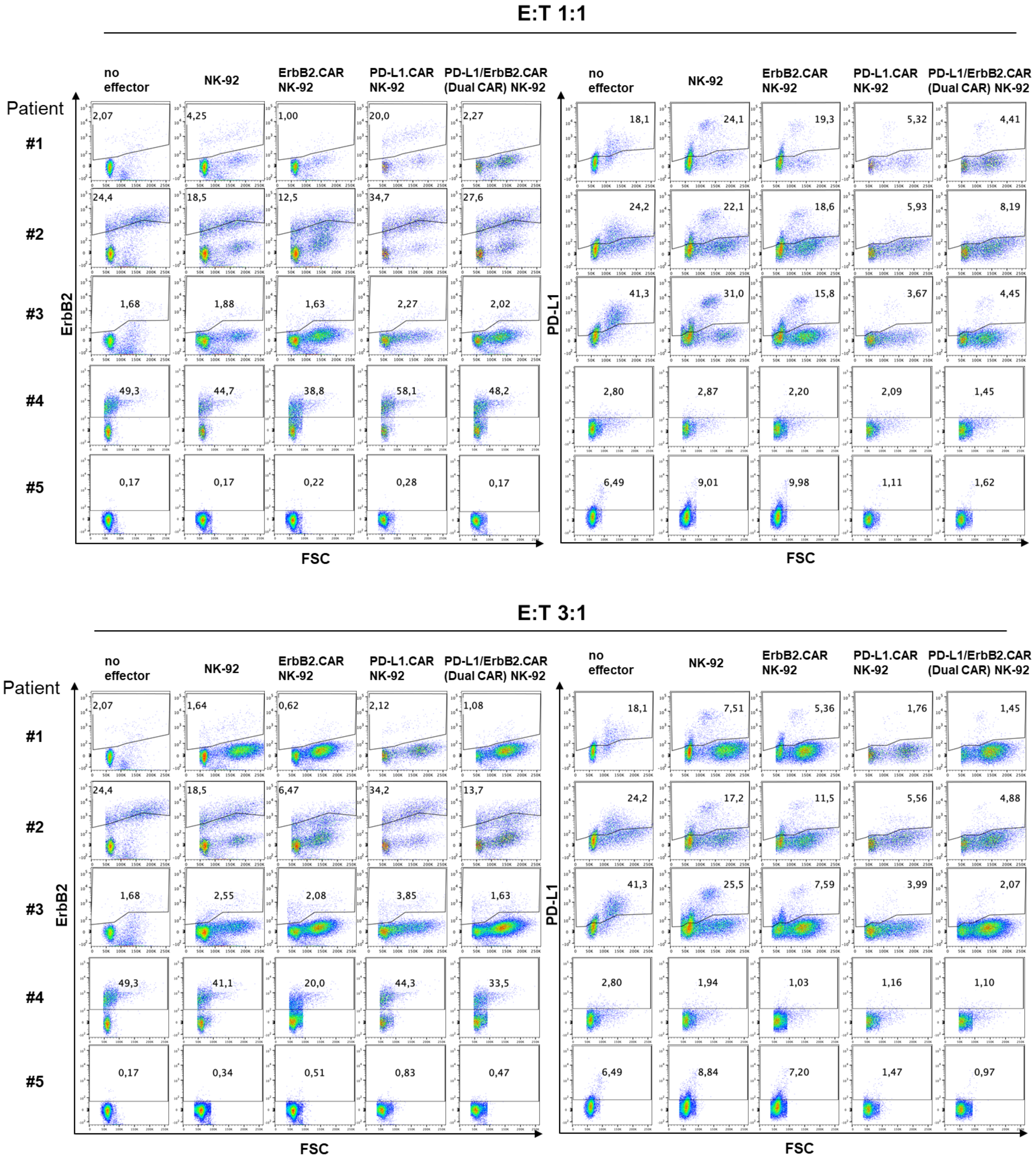

E:T 1:1
Patient
#1
#2
#3
#4
#5
no effector
NK-92
ErbB2.CAR NK-92
PD-L1.CAR NK-92
PD-L1/ErbB2.CAR (Dual CAR) NK-92
ErbB2
PD-L1
FSC
2,07
4,25
1,00
20,0
2,27
24,4
18,5
12,5
34,7
27,6
1,68
1,88
1,63
2,27
2,02
49,3
44,7
38,8
58,1
48,2
0,17
0,17
0,22
0,28
0,17
18,1
24,1
19,3
5,32
4,41
24,2
22,1
18,6
5,93
8,19
41,3
31,0
15,8
3,67
4,45
2,80
2,87
2,20
2,09
1,45
6,49
9,01
9,98
1,11
1,62
E:T 3:1
2,07
1,64
0,62
2,12
1,08
24,4
18,5
6,47
34,2
13,7
1,68
2,55
2,08
3,85
1,63
49,3
41,1
20,0
44,3
33,5
0,17
0,34
0,51
0,83
0,47
18,1
7,51
5,36
1,76
1,45
24,2
17,2
11,5
5,56
4,88
41,3
25,5
7,59
3,99
2,07
2,80
1,94
1,03
1,16
1,10
6,49
8,84
7,20
1,47
0,97

Fig. S18

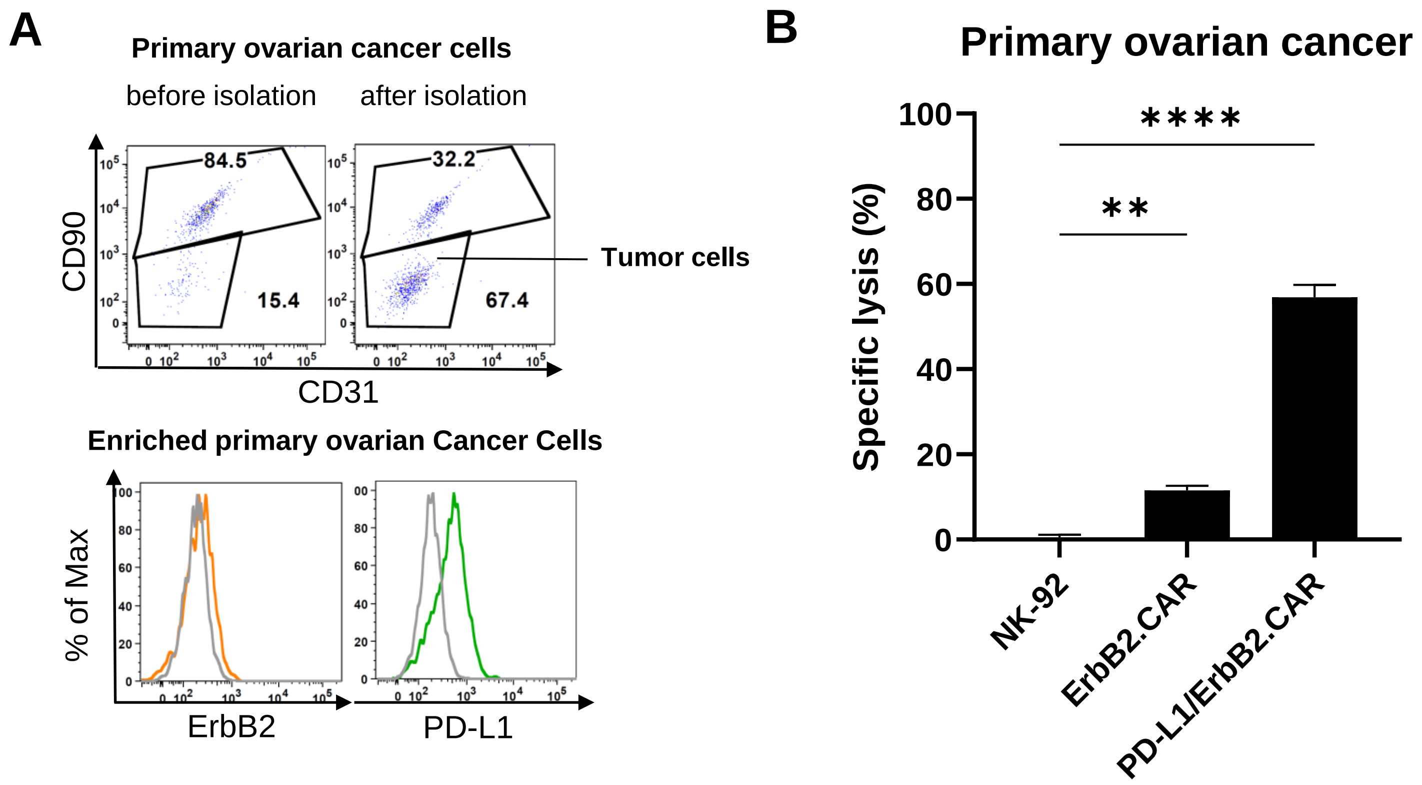
A
Primary ovarian cancer cells
before isolation
after isolation
84.5
15.4
32.2
67.4
CD90
CD31
Tumor cells
Enriched primary ovarian Cancer Cells
% of Max
ErbB2
PD-L1
B
Primary ovarian cancer
Specific lysis (%)
100
80
60
40
20
0
****
**
NK-92
ErbB2.CAR
PD-L1/ErbB2.CAR

Fig. S19

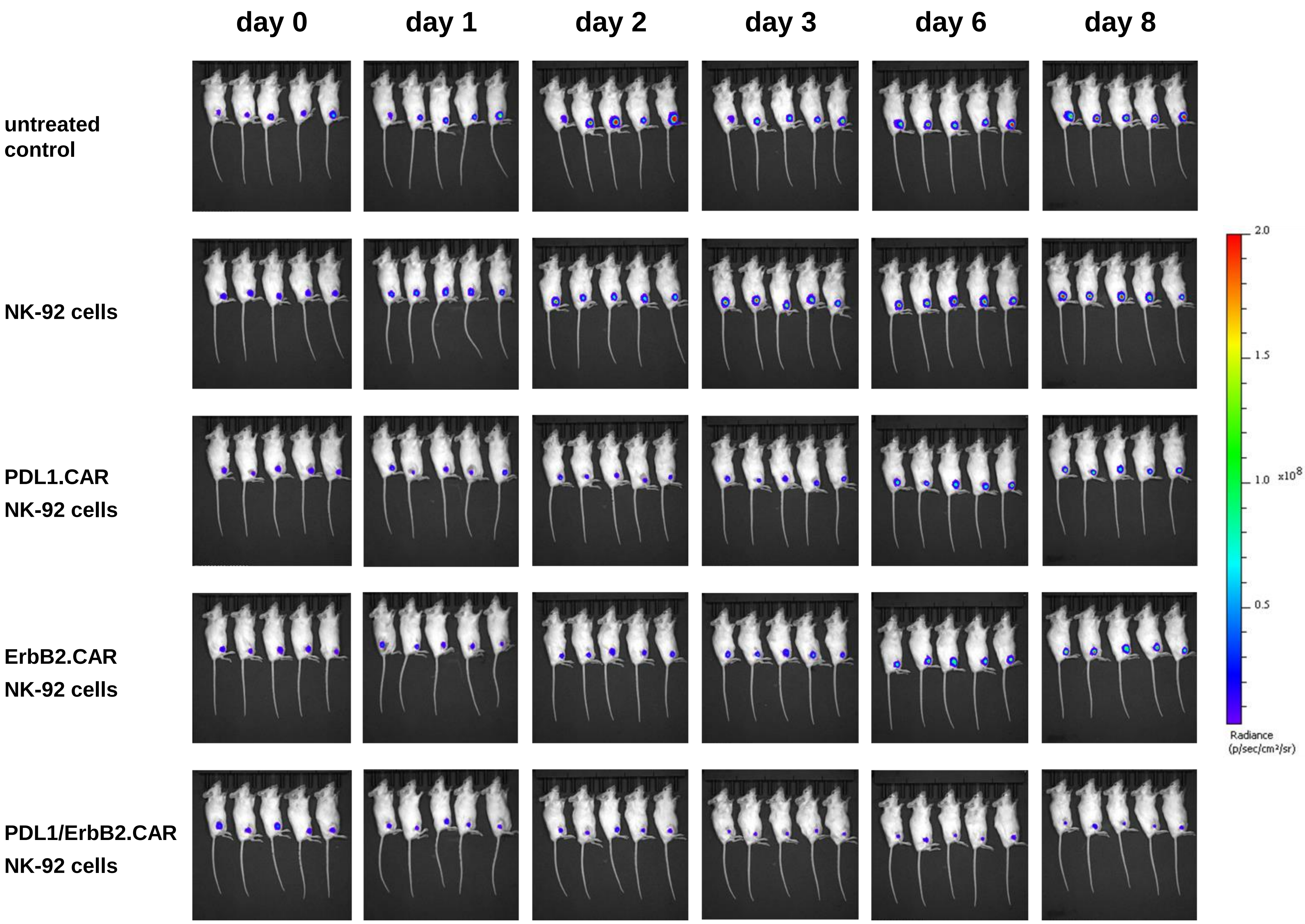
day 0
day 1
day 2
day 3
day 6
day 8
untreated control
NK-92 cells
PDL1.CAR NK-92 cells
ErbB2.CAR NK-92 cells
PDL1/ErbB2.CAR NK-92 cells
2.0
1.5
1.0
x10^8
0.5
Radiance (p/sec/cm²/sr)
